# Mitogenomic phylogenies suggest the resurrection of the subfamily Porrocaecinae and provide insights into the systematics of the superfamily Ascaridoidea (Nematoda: Ascaridomorpha), with the description of a new species of *Porrocaecum*

**DOI:** 10.1186/s13071-023-05889-9

**Published:** 2023-08-10

**Authors:** Xiao-Hong Gu, Ning Guo, Hui-Xia Chen, Jiljí Sitko, Lin-Wei Li, Bing-Qian Guo, Liang Li

**Affiliations:** 1 Hebei Key Laboratory of Animal Physiology, Biochemistry and Molecular Biology, Hebei Collaborative Innovation Center for Eco-Environment, College of Life Sciences, Hebei Normal University, Shijiazhuang, 050024 Hebei Province People’s Republic of China; 2 Hebei Research Center of the Basic Discipline Cell Biology, Ministry of Education Key Laboratory of Molecular and Cellular Biology, Shijiazhuang, 050024 Hebei Province People’s Republic of China; 3 Muzeum Komenského V Přerově, 750 02 Přerově, Czech Republic

**Keywords:** Parasitic nematodes, Ascaridomorpha, Birds, Integrated taxonomy, Mitochondrial genome, Phylogeny, New species

## Abstract

**Background:**

The family Toxocaridae is a group of zooparasitic nematodes of veterinary, medical and economic significance. However, the evolutionary relationship of *Porrocaecum* and *Toxocara*, both genera currently classified in Toxocaridae, and the monophyly of the Toxocaridae remain under debate. Moreover, the validity of the subgenus *Laymanicaecum* in the genus *Porrocaecum* is open to question. Due to the scarcity of an available genetic database, molecular identification of *Porrocaecum* nematodes is still in its infancy.

**Methods:**

A number of *Porrocaecum* nematodes collected from the Eurasian marsh harrier *Circus aeruginosus* (Linnaeus) (Falconiformes: Accipitridae) in the Czech Republic were identified using integrated morphological methods (light and scanning electron microscopy) and molecular techniques (sequencing and analyzing the nuclear 18S, 28S and ITS regions). The complete mitochondrial genomes of the collected nematode specimens and of *Porrocaecum* (*Laymanicaecum*) *reticulatum* (Linstow, 1899) were sequenced and annotated for the first time. Phylogenetic analyses of ascaridoid nematodes based on the amino acid sequences of 12 protein-coding genes of mitochondrial genomes were performed using maximum likelihood and Bayesian inference.

**Results:**

A new species of *Porrocaecum*, named *P. moraveci* n. sp., is described based on the morphological and genetic evidence. The mitogenomes of *P. moraveci* n. sp. and *P. reticulatum* both contain 36 genes and are 14,517 and 14,210 bp in length, respectively. Comparative mitogenomics revealed that *P.* *moraveci* n. sp. represents the first known species with three non-coding regions and that *P. reticulatum* has the lowest overall A + T content in the mitogenomes of ascaridoid nematodes tested to date. Phylogenetic analyses showed the representatives of *Toxocara* clustered together with species of the family Ascarididae rather than with *Porrocaecum* and that *P. moraveci* n. sp. is a sister to *P. reticulatum*.

**Conclusions:**

The characterization of the complete mitochondrial genomes of *P. moraveci* n. sp. and *P. reticulatum* is reported for the first time. Mitogenomic phylogeny analyses indicated that the family Toxocaridae is non-monophyletic and that the genera *Porrocaecum* and *Toxocara* do not have an affinity. The validity of the subgenus *Laymanicaecum* in *Porrocaecum* was also rejected. Our results suggest that: (i) Toxocaridae should be degraded to a subfamily of the Ascarididae that includes only the genus *Toxocara*; and (ii) the subfamily Porrocaecinae should be resurrected to include only the genus *Porrocaecum*. The present study enriches the database of ascaridoid mitogenomes and provides a new insight into the systematics of the superfamily Ascaridoidea.

**Graphical Abstract:**

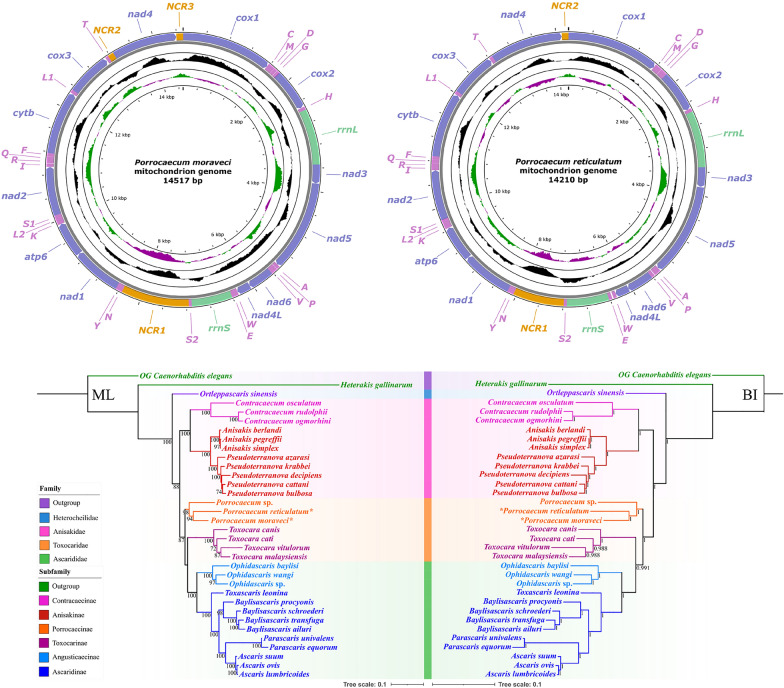

**Supplementary Information:**

The online version contains supplementary material available at 10.1186/s13071-023-05889-9.

## Background

The superfamily Ascaridoidea comprises a large group of parasitic nematodes that commonly occur in the digestive tract of all major lineages of vertebrates [1–6]. The Ascaridoidea is currently divided into six major families, namely Heterocheilidae, Acanthocheilidae, Anisakidae, Ascarididae, Toxocaridae and Raphidascarididae [7]. Among them, the family Toxocaridae (Ascaridomorpha: Ascaridoidea) contains only two genera, *Porrocaecum* and *Toxocara* [4, 8, 9], with over 50 nominal species parasitizing birds and mammals worldwide [1, 2, 10]. Nematodes of the family Toxocaridae cause diseases in wildlife, domestic animals and humans and are therefore of veterinary, medical and economic significance [2, 11–13]. However, the evolutionary relationship of *Porrocaecum* and *Toxocara*, and the monophyly of the Toxocaridae remain under debate. Results from a number of earlier phylogenetic studies indicated that *Porrocaecum* and *Toxocara* have no close relationship and that Toxocaridae is not monophyletic [14–17] while, in contrast, the results of another molecular phylogeny study supported the monophyly of the Toxocaridae and showed an affinity between *Porrocaecum* and *Toxocara* [7].

Nematodes of the genus *Porrocaecum* are common parasites that mainly occur in the digestive tract of various species of birds worldwide [2, 18–21]. In 1953, Mozgovoi proposed dividing the genus *Porrocaecum* into two subgenera, *Laymanicaecum* and *Porrocaecum*, based on the presence or absence of the gubernaculum in the male [2]. However, this proposal was rejected by Hartwich [4]. To date, the validity of the subgenus *Laymanicaecum* has never been tested based on molecular phylogeny due to the scarcity and inaccessibility of suitable material or genetic data.

Although approximately 40 species of *Porrocaecum* have been described, the validity of some species is still questionable due to their high morphological similarities [22]. Moreover, molecular identification of *Porrocaecum* nematodes using various nuclear and mitochondrial DNA (mtDNA) markers [large ribosomal DNA (28S), internal transcribed spacer (ITS) and cytochrome *c* oxidase subunit 1 (*cox*1) or 2 (*cox*2)] remains in its infancy due to a scarcity of available genetic databases. To date, there have been only eight species of *Porrocaecum* with their genetic data recorded in the GenBank database [7, 22]. Among these, only one unidentified species, *Porrocaecum* sp., has been sequenced for the complete mitochondrial genome [14].

In the present study, a number of *Porrocaecum* nematodes were collected from the Eurasian marsh harrier (*Circus aeruginosus* (Linnaeus); Falconiformes: Accipitridae) in the Czech Republic. In order to accurately identify these *Porrocaecum* nematodes to species level, we observed the detailed morphology of the present specimens using light and scanning electron microscopy, and the nuclear 18S, 28S and ITS regions were sequenced and analyzed. The complete mitochondrial genomes of the collected *Porrocaecum* nematodes and a representative of the subgenus *Laymanicaecum*, *Porrocaecum* (*Laymanicaecum*) *reticulatum* (Linstow, 1899), were also sequenced and annotated for the first time to reveal the patterns of mitogenomic evolution in this group. Moreover, in order to test the monophyly of the Toxocarinae/Toxocaridae and determine the systematic status of the subgenus *Laymanicaecum* in *Porrocaecum*, phylogenetic analyses of ascaridoid nematodes based on the amino acid sequences of 12 protein-coding genes (PCGs) of mitochondrial genomes and phylogeny of *Porrocaecum* based on 18S + ITS and 28S were performed using maximum likelihood (ML) and Bayesian inference (BI), respectively.

## Methods

### Parasite collection and species identification

Nematode specimens of *Porrocaecum* were collected from the intestine of the Eurasian marsh harrier *C. aeruginosus* (Falconiformes: Accipitridae) during a helminthological survey of birds in Czech Republic. The collected specimens were washed in saline, then stored in 70% ethanol until studied. For the light microscopy studies, nematodes were cleared in lactophenol, and drawings were made with the aid of a Nikon microscope drawing attachment (Nikon Corp., Tokyo, Japan). For the scanning electron microscopy (SEM) studies, specimens were post-fixed in 1% OsO4, dehydrated through an ethanol and acetone series and then critical point dried. The specimens were then coated with gold and examined using a Hitachi S-4800 scanning electron microscope at an accelerating voltage of 20 kV (Hitachi Ltd., Tokyo, Japan). In this article, measurements (the range, with the mean in parentheses) are presented in micrometers unless otherwise stated. For study of the mitochondrial genome, specimens of *P.* (*Laymanicaecum*) *reticulatum* were collected from the great egret [*Ardea alba* (Linnaeus); Ciconiiformes: Ardeidae] in Hustopeče and Bečvou, Czech Republic.

### Molecular procedures

The mid-body of two nematode specimens (1 male, 1 female) was used for molecular analyses. Genomic DNA from each sample was extracted using a Column Genomic DNA Isolation Kit [Sangon Biotech （Shanghai） Co., Ltd., Shanghai, China] according to the manufacturer's instructions. The primers used for amplifying the target sequences of 18S, ITS and 28S were: primers 18SF and 18SR for the partial 18S [23]; primers SS1 and SS2R for the partial ITS region ITS-1 region [24]; primers NC13 and NC2 for ITS-2 [24]; and primers 28SF and 28SR for the partial 28S ribosomal DNA (rDNA) [15]. The cycling conditions were as described previously [7]. PCR products were checked on GoldView-stained 1.5% agarose gels and purified with Column PCR Product Purification Kit [Sangon Biotech （Shanghai） Co., Ltd.]. Sequencing of each sample was carried out for both strands. Specifically, sequences were aligned using ClustalW2. The DNA sequences obtained herein were compared (using the algorithm BLASTn) with those available in the National Center for Biotechnology Information (NCBI) database (http://www.ncbi.nlm.nih.gov). The 18S, 28S and ITS sequence data obtained herein have been deposited in the GenBank database (http://www.ncbi.nlm.nih.gov).

### Mitochondrial genome sequencing, assembly and annotation

A total of 30 Gb of clean genomic data of each species was generated using the Pair-End 150 sequencing method on the Illumina NovaSeq 6000 platform (Illumina, Inc., San Diego, CA, USA) by Novogene Technology Co., Ltd. (Tianjin, China). The complete mitochondrial genome was assembled using GetOrganelle v1.7.2a [25]. PCGs, ribosomal RNAs (rRNAs) and transfer RNAs (tRNAs) were annotated using the MitoS web server (http://mitos2.bioinf.uni-leipzig.de/index.py) and the MitoZ v2.4 toolkit [26]. The open reading frame (ORF) of each PCG was confirmed manually through the web version of ORF finder (https://www.ncbi.nlm.nih.gov/orffinder/). The “lost” tRNA genes ignored by both MitoS and MitoZ were identified using BLAST based on a database of the existing tRNA sequences of nematodes (CNP0003131, NC_010690, NC_070176). The secondary structures of tRNAs were predicted by the ViennaRNA module [27], building on MitoS2 [28] and the RNAstructure v6.3 software package [29], followed by a manual correction. The MitoZ v2.4 toolkit was used to visualize and depict gene element features [26]. The base composition, amino acid usage and relative synonymous codon usage (RSCU) were calculated by Python script, which refers to the Codon Adaptation Index (CAI) [30]. The total length of the base composition included ambiguous bases. Base skew analysis was used to describe the base composition of nucleotide sequences. The complete mitochondrial genomes of *P. moraveci* n. sp. and *P. reticulatum* obtained herein were deposited in the GenBank database (http://www.ncbi.nlm.nih.gov).

### Phylogenetic analyses

Phylogenetic analyses of ascaridoid nematodes were performed based on the amino acid sequences of 12 PCGs of mitochondrial genomes using ML and BI, respectively. *Caenorhabditis elegans* (Rhabditida: Rhabditoidea) and *Heterakis gallinarum* (Ascaridomorph: Heterakoidea) were chosen as the outgroup. The ingroup included 32 representatives of the superfamily Ascaridoidea. Detailed information on the representatives included in the present phylogeny analysis is provided in Table 1. The phylogenetic trees were re-rooted on *C. elegans*. Genes were aligned separately using the MAFFT v7.313 multiple sequence alignment program under the iterative refinement method of E-INS-I [31]. Ambiguous sites and poorly aligned positions were eliminated using the BMGE v1.12 program (m = BLOSUM90, h = 0.5) [32]. The aligned and eliminated sequences were concatenated into a matrix by the PhyloSuite v1.2.2 desktop platform [33]. The mtMet + F + R4 model was identified as the optimal nucleotide substitution model for the ML inference. The partitioning schemes and the optimal nucleotide substitution model selected for each combination of partition for the BI inference are shown in Additional file 1: Table S1. Reliabilities for ML inference were tested using 1000 bootstrap (BS) replications, and BIC analysis was run for 5 × 10^6^ Markov chain Monte Carlo (MCMC) generations.

**Table 1:** Detailed information on the representatives of Ascaridoidea included in the present phylogeny study

Species	Mitochondrial genomes	Length (bp)	A+T content (%)	References
*Outgroup*				
*Caenorhabditis elegans*	NC_001328	13,794	76.2	[58]
*Heterakis gallinarum*	NC_029839	13,973	69.84	[59]
*Ingroup*				
Anisakidae				
*Anisakis berlandi*	NC_026023	13,915	71.25	Unpublished
*Anisakis pegreffii*	NC_034329	14,002	71.36	[47]
*Anisakis simplex*	KU899549	13,938	71.32	Unpublished
*Contracaecum ogmorhini*	NC_031647	14,019	71.37	Unpublished
*Contracaecum osculatum*	NC_024037	13,823	70.22	[45]
*Contracaecum rudolphii*	NC_014870	14,022	70.45	Unpublished
*Pseudoterranova azarasi*	NC_027163	13,954	70.71	[60]
*Pseudoterranova bulbosa*	NC_031643	13,957	71.24	Unpublished
*Pseudoterranova cattani*	NC_031644	13,950	71.08	Unpublished
*Pseudoterranova decipiens*	NC_031645	13,962	71.04	Unpublished
*Pseudoterranova krabbei*	NC_031646	13,948	70.43	Unpublished
Ascarididae				
* Ascaris lumbricoides*	NC_016198	14,281	71.82	[61]
*Ascaris ovis*	MT993838	14,205	71.96	[62]
*Ascaris suum*	NC_001327	14,284	71.97	[58]
*Baylisascaris ailuri*	NC_015925	14,657	69.47	[63]
*Baylisascaris procyonis*	JF951366	14,781	70.46	[64]
*Baylisascaris schroederi*	HQ671081	14,778	68.62	[63]
*Baylisascaris transfuga*	HQ671079	14,898	69.45	[63]
*Parascaris equorum*	NC_036427	13,899	70.25	[49]
*Parascaris univalens*	NC_024884	13,920	70.57	[50]
*Toxascaris leonina*	MK516267	14,685	71.06	[65]
*Ophidascaris* sp.	CNA0050675	14,641	70.23	[14]
*Ophidascaris wangi*	MK106624	14,660	69.24	[66]
*Ophidascaris baylisi*	MW880927	14,784	69.98	[48]
Heterocheilidae				
*Ortleppascaris sinensis*	KU950438	13,828	74.02	[67]
Toxocaridae				
* Toxocara canis*	NC_010690	14,322	68.57	[40]
*Toxocara cati*	NC_010773	14,029	69.95	[40]
*Toxocara malaysiensis*	NC_010527	14,266	68.86	[40]
*Toxocara vitulorum*	NC_070176	15,045	69.95	[17]
*Porrocaecum* sp.	CNA0050678	14,568	71.42	[14]
*Porrocaecum moraveci*	OQ863051	14,517	69.95	Present study
*Porrocaecum reticulatum*	OQ863050	14,210	67.22	Present study

Phylogenetic analyses of *Porrocaecum* species were performed based on the 18S + ITS and 28S sequence data using the ML method with IQTREE v2.1.2 [34] and BI with MrBayes 3.2.7 [35], respectively. *Toxocara cati* (Ascaridida: Ascaridoidea) was chosen as the out-group. Detailed information on the *Porrocaecum* species included in the present phylogeny analysis is provided in Table 2. Three partitions and their models were selected for ML analyses: K2P + FQ + I (18S); K2P + FQ + G4 (ITS-1 + 5.8S + ITS-2); and TPM3 + F + G4 (28S). Similarly, three partitions were used for BI analyses: K80 + I (18S); K80 + G (ITS-1 + 5.8S + ITS-2); and HKY + G (28S). Reliabilities for ML inference were tested using 1000 BS replications, and BIC analysis was run for 5 × 10^6^ MCMC generations.

**Table 2:** Species of Porrocaecinae with detailed genetic information included in the phylogenetic analyses

Species	Host	Locality	GenBank ID for 18S region	GenBank ID for ITS region	GenBank ID for 28S region	References
*Ingroup*						
*P. moraveci*	*Circus aeruginosus* (Falconiformes: Accipitridae)	Czech Republic	OQ858491, OQ858492	OQ858560, OQ858561	OQ858562, OQ858563	Present study
*P. reticulatum*	*Ardea alba* (Ciconiiformes: Ardeidae)	Czech Republic	OQ851895	OQ857284	OQ863745	Present study
*P. reticulatum*	*Egretta garzetta* (Pelecaniformes: Ardeidae)	China	MF072700	MF061688	—	[7]
*P. ensicaudatum*	*Turdus americanus* (Passeriformes: Turdidae); *Sturnus vulgaris* (Passeriformes: Sturnidae)	USA; Czech Republic	KX172116	AY603532	—	Unpublished; [68]
*P. depressum*	*Strix varia* (Strigiformes: Strigidae); *Buteo buteo* (Accipitriformes: Accipitridae)	USA; Czech Republic	U94379	AY603534	U94765	[15]; [68]
*P. streperae*	*Gymnorhina tibicen* (Passeriformes: Artamidae)	Australia	EF180074	AJ007460, Y09497	–	[69]; [70]
*P. angusticolle*	*Accipiter nisus* (Accipitriformes: Accipitridae); *Buteo buteo* (Accipitriformes: Accipitridae)	Germany; Czech Republic	EU004820	MW447303	MW441213–MW441216	[71]; [22]
*Outgroup*						
*Toxocara cati*	*Felis domesticus* (Carnivora: Felidae); *Prionailurus bengalensis* (Carnivora: Felidae)	USA; China	EF180059	KY003067	JN256993	[69, 72]; [73]

*ITS* Internal transcribed spacer,* 18S/28S* small/large ribosomal subunit

In the ML tree, BS values ≥ 90 were considered to constitute strong branch support, whereas bootstrap values ≥ 70 and < 90 were considered to constitute moderate branch support. In the BI tree, Bayesian posterior probabilities (BPP) values ≥ 0.90 were considered to constitute strong branch support, whereas BPP values ≥ 0.70 and < 0.90 were considered to constitute moderate branch support. BS values ≥ 70 and BPP values ≥ 0.70 are shown in the phylogenetic trees.

## Results

**Superfamily Ascaridoidea RailIiet & Henry, 1912**

**Family Ascarididae Baird, 1853**

**Subfamily Porrocaecinae Osche, 1958**

**Genus** ***Porrocaecum*** **Railliet & Henry, 1912**

***Porrocaecum* (*Porrocaecum*)** ***moraveci* sp. n.**

**Type-host**: *Circus aeruginosus* (Linnaeus) (Falconiformes: Accipitridae).

**Type-locality**: Přerov, Czech Republic.

**Site in host**: Intestine.

**Type specimens**: Holotype, male (HBNU–N–B20220021GL); allotype, female (HBNU–N–B20220022GL); paratype: 1 male (HBNU–N–B20220023GL); deposited in the College of Life Sciences, Hebei Normal University, Hebei Province, China.

**Representative DNA sequences**: Representative nuclear ribosomal and mitochondrial genome sequences were deposited in the GenBank database under the accession numbers OQ858491, OQ858492 (18S), OQ858562, OQ858563 (28S), OQ858560, OQ858561 (ITS), OQ863051 (mitochondrial genome).

**ZooBank registration**: To comply with the regulations set out in article 8.5 of the amended 2012 version of the International Code of Zoological Nomenclature (ICZN), details of the new species have been submitted to ZooBank. The Life Science Identifier (LSID) of the article is urn: lsid: zoobank.org: pub: 6F8AE7EE-67E8-41BF-AB2C-4EA4874D8843. The LSID for the new name *Porrocaecum moraveci* is urn: lsid: zoobank.org: act: 09174C82-DAF4-4C78-B47B-AA82C9B8FC74.

**Etymology**: The species is named in honor of Dr. František Moravec (Institute of Parasitology, Biology Centre of the Czech Academy of Sciences, Czech Republic), who has made great contributions to the taxonomy of ascaridoid nematodes.

### Description

#### General description

**Fig. 1:**
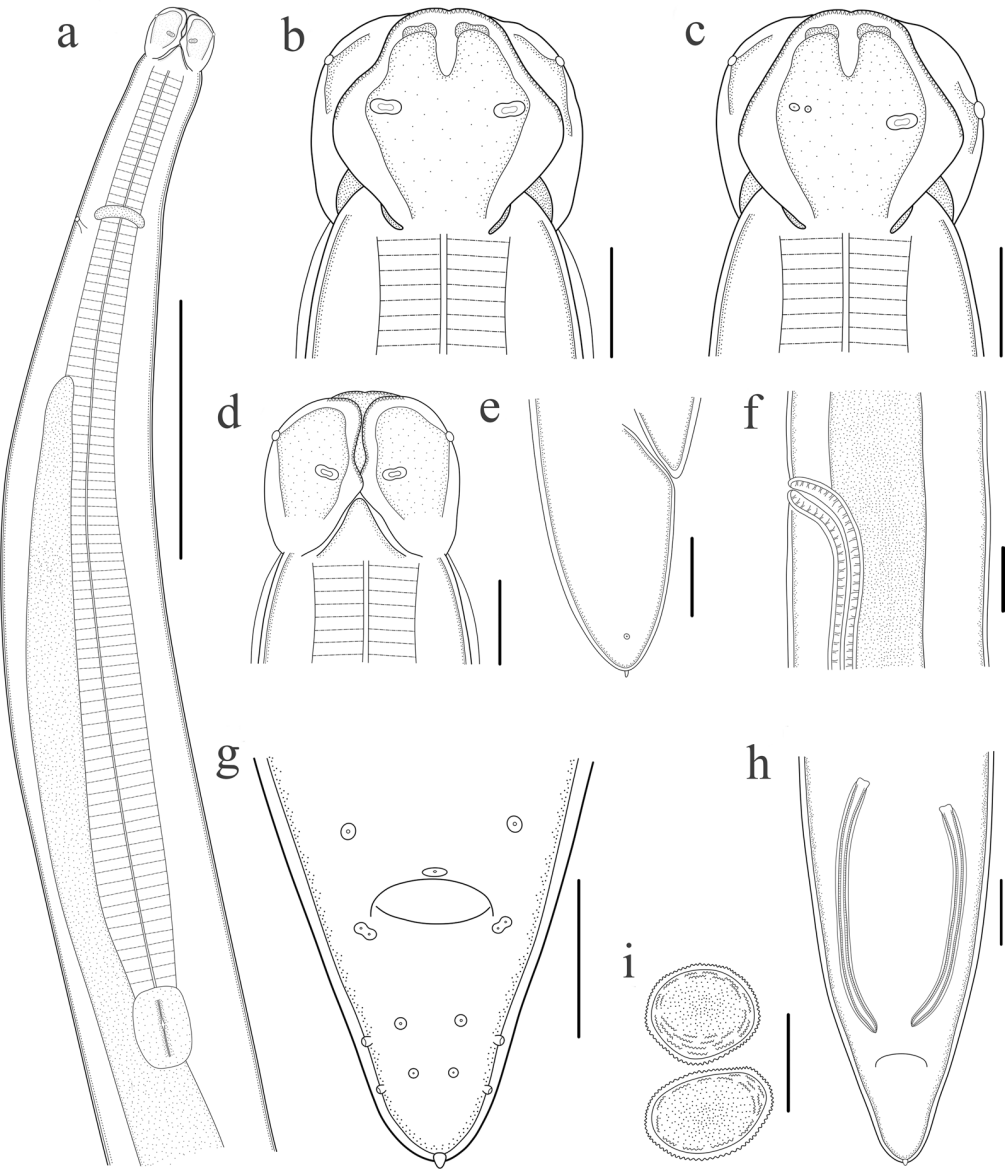
*Porrocaecum moraveci* n. sp. from *Circus aeruginosus* in Czech Republic.** a** anterior part of male body, lateral view;** b** cephalic end of male, dorsal view;** c** cephalic end of male, subventral view;** d** cephalic end of male, ventral view;** e** tail of female, lateral view;** f** region of vulva, lateral view;** g** tail of male, ventral view;** h** posterior end of male (showing spicules), ventral view;** i** eggs. Scale bars:** a**, 1000 μm;** b**–**d**,** i**, 100 μm;** e**,** g**,** h**, 200 μm;** f**, 300 μm

**Fig. 2:**
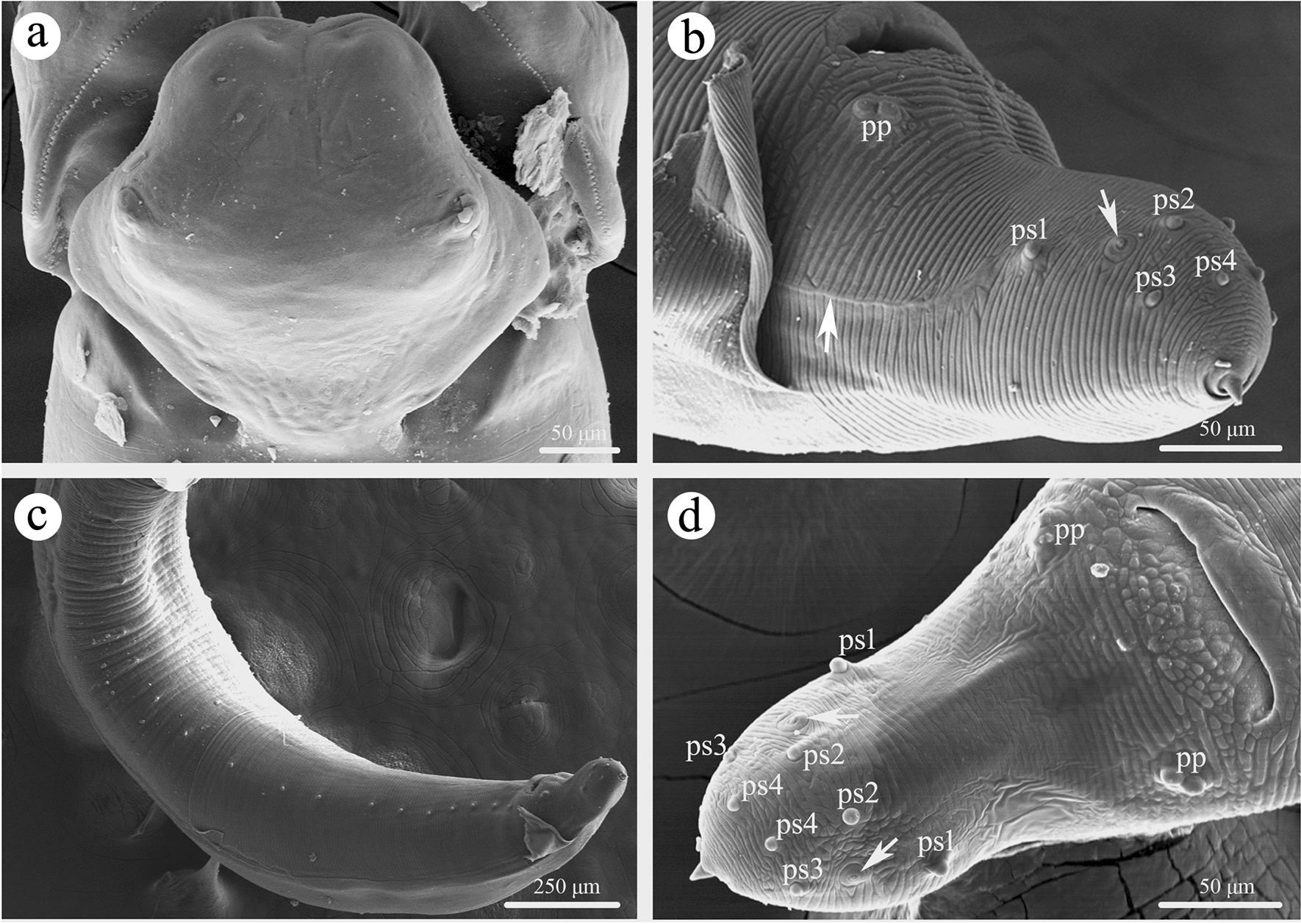
Scanning electron micrographs of *Porrocaecum moraveci* n. sp. collected from *Circus aeruginosus* in Czech Republic, male. **a** dorsal lip; **b** tail (arrow indicates lateral alae and phasmid), lateral view; **c** posterior end of body, lateral view; **d** tail (arrow indicates phasmid), ventral view. pp, paracloacal papillae; ps, postcloacal papillae

**Fig. 3:**
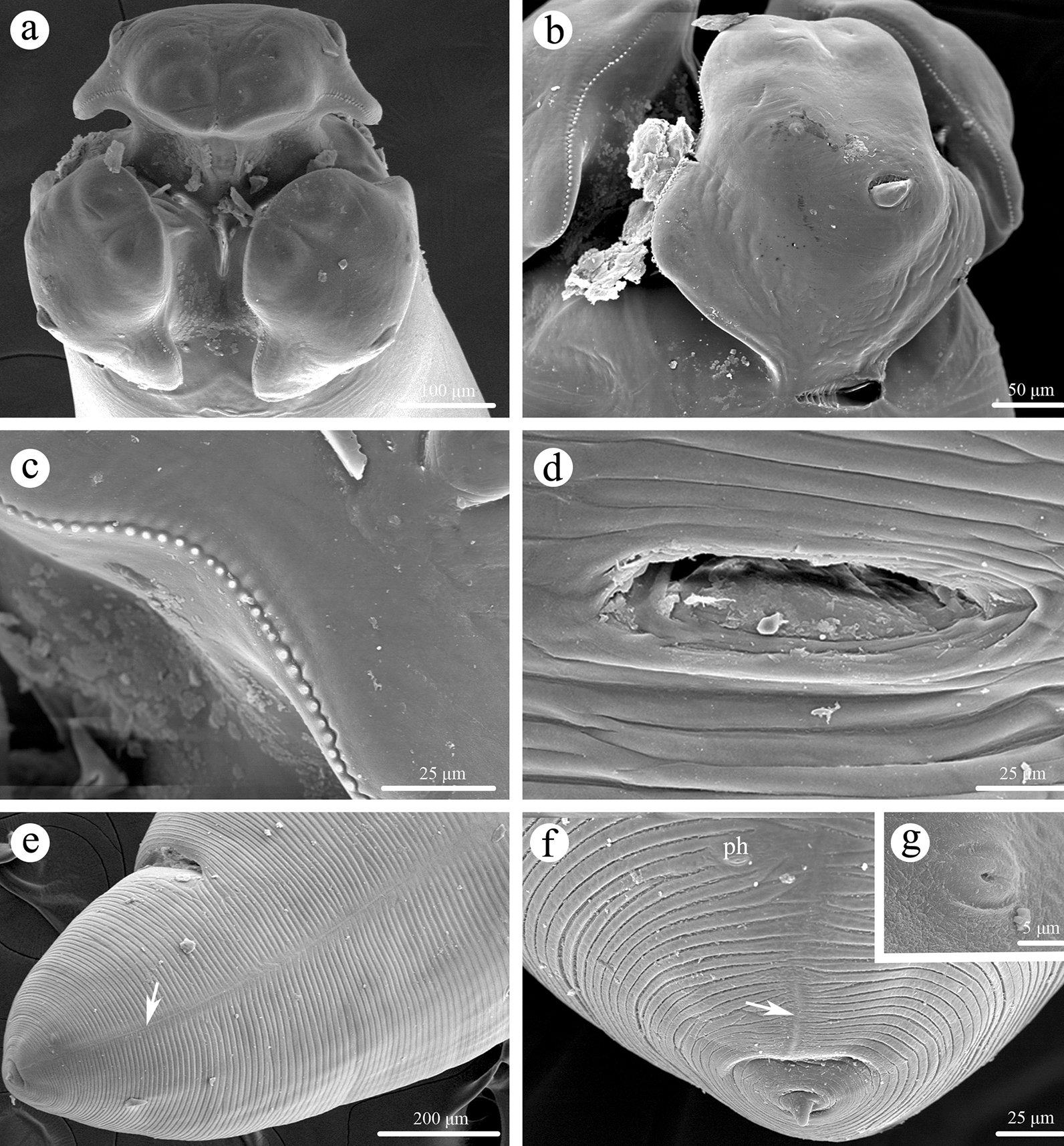
Scanning electron micrographs of *Porrocaecum moraveci* n. sp. collected from *Circus aeruginosus* in Czech Republic, female. **a** cephalic end, apical view; **b** ventro-lateral lip; **c** magnified image of labial denticles; **d** magnified image of vulva; **e** tail (arrow indicates lateral ala), lateral view; **f** magnified image of tail tip (arrow indicates lateral ala), lateral view; **g** magnified image of phasmid. ph, phasmid

 Large-sized, whitish nematodes with transversely striated cuticle. Maximum width at about mid-body. Anterior extremity with three roughly hexagonal lips, postlabial grooves and lateral membranous flanges conspicuous (Figs. 1a–d, 2a, 3a, b). Dorsal lip with one pair of large double papillae (Figs. 1b, 2a); subventral lips each with single double papilla, small papilla and amphid (Figs. 1c, 3c). Single median superficial ditch and pair of small, submedial pores present on each lip (Figs. 2a, 3a, b). Anterior and lateral margins of each lip armed with about 100–120 acuminate denticles (Figs. 2a, 3b, c). Interlabia small, triangular, about one third of length of lips (Figs. 1a, d, 2a, 3a, b). Cervical alae absent. Cervical papillae not observed. Esophagus muscular, distinctly broader posteriorly than anteriorly (Fig. 1a). Ventriculus longer than wide (Fig. 1a). Intestinal caecum long, about two thirds of esophageal length (Fig. 1a). Ventricular appendix absent. Nerve-ring at about one fifth of esophageal length. Excretory pore just posterior to the nerve-ring (Fig. 1a). Tail of both sexes conical, with a very small finger-like mucron (Figs. 1e, g, h, 2b, d, 3f).

#### Male

 Based on one mature specimen. Body 62.0 mm long; maximum width 781 μm. Dorsal lip 213 μm long, 203 μm wide. Interlabia 73 μm long. Esophagus 3.61 μm long, 300 μm in maximum width, representing 5.80% of body length. Nerve-ring and excretory pore 820 and 855 μm, respectively, from anterior extremity. Ventriculus 290 μm long, 210 μm wide. Intestinal caecum 2.66 mm long, 235 μm wide, representing 73.7% of esophageal length. Posterior end of body distinctly curved ventrally. Spicules alate, unequal in length, sub-rounded at distal end, left spicule 792 μm long, representing 1.30% of body length; right spicule 696 μm long, representing 1.10% of body length (Fig. 1h). Gubernaculum absent. Caudal papillae 25 pairs in total: 20 pairs precloacal, one pair double paracloacal (slightly posterior to cloaca) and four pairs postcloacal (2 pairs ventral, 2 pairs lateral) (Figs. 1g, 2b–d). Single medio-ventral precloacal papilla unconspicuous (Fig. 2d). Tail 330 μm long. Caudal alae weak (Fig. 2b). Lateral phasmids present (Fig. 2, d).

#### Female

Based on two mature specimens (measurements presented as the range with the mean in parentheses). Body 117.0–138.0 (127.5) mm long; maximum width 1.39–1.44 (1.41) mm. Dorsal lip 295–375 (335) μm long, 365–510 (438) μm wide. Interlabia 114–116 (115) μm long. Esophagus 4.68–6.61 (5.65) mm long, 490–510 μm (500) in maximum width, representing 4.00–4.80 (4.40)% of body length. Nerve-ring and excretory pore 970–1140 (1055) μm and 1.12–1.36 (1.24) mm, respectively, from anterior extremity. Ventriculus 330–450 (390) μm long, 240–380 (310) μm wide. Intestinal caecum 2.85–4.52 (3.69) mm long, 210–230 (220) μm in maximum width, representing 60.9–68.5 (64.7)% of esophageal length. Vulva slit-like, pre-equatorial, 36.8–43.5 (40.2) mm from anterior extremity, at 31.5% of body length (Figs. 1f, 3d). Vagina muscular, directed posteriorly from vulva. Eggs oval, thick-shelled, with punctate surface, 105–165 (129) × 80–120 (99) μm (*n* = 25) (Fig. 1i). Tail 500–598 (549) μm long (Figs. 1f, 3e). Caudal alae weak (Fig. 3e, f). Lateral phasmids present (Figs. 1e, 2f, g).

### Genetic characterization

#### Partial 18S region

Two 18S sequences of *P. moraveci* sp. n. obtained herein are 1717 bp in length, with no nucleotide divergence detected. In the genus *Porrocaecum*, the 18S sequence data are available in GenBank for P*. angusticolle* (Molin, 1860) (EU004820), *P. depressum* (Zeder, 1800) (U94379), *P. reticulatum* (Linstow, 1899) (MF072700), *Porrocaecum* sp. (MT141136) and *P. streperae* Johnston & Mawson, 1941 (EF180074). Pairwise comparison of the 18S sequences of *P. moraveci* with those of *Porrocaecum* spp. showed 0.17% (*Porrocaecum* sp.) to 0.47% (*P. depressum* and *P. streperae*) of nucleotide divergence.

#### Partial 28S region

Two 28S sequences of *P. moraveci* sp. n. obtained herein are 746 bp in length, with no nucleotide divergence detected. In the genus *Porrocaecum*, the 28S sequences are only available in GenBank for *P. angusticolle* (MW441213-MW4412136) and *P. depressum* (U94765). Pairwise comparison of the 28S sequences of *P. moraveci* with those of *P. angusticolle* and *P. depressum* showed 1.88% (*P. angusticolle*) and 10.8% (*P. depressum*) of nucleotide divergence.

#### Partial ITS (ITS-1 + 5.8S + ITS-2) region

Two *ITS* sequences of *P. moraveci* sp. n. obtained herein are 988 bp in length, with no nucleotide divergence detected. In the genus *Porrocaecum*, the ITS-1 + 5.8S + ITS-2 sequences are available in GenBank for *P. angusticolle* (MW447303–MW447305), *P. crassum* (Deslongchamps, 1824) (AY603533), *P. depressum* (AY603534), *P. ensicaudatum* (Zeder, 1800) (AY603532), *P. reticulatum* (MF061688), *P. streperae* (AJ007460) and *Porrocaecum* sp. (LC666446). Pairwise comparison of the ITS-1 + 5.8S + ITS-2 sequences of *P. moraveci* with those of *P. angusticolle*, *P. crassum*, *P. depressum*, *P. ensicaudatum*, *P. reticulatum*, *P. streperae* and *Porrocaecum* sp. showed 8.30% (*P. angusticolle*) to 30.1% (*P. crassum*) of nucleotide divergence.

### Remarks

We assigned the present specimens to the genus *Porrocaecum* based on the combination of morphological characters, including the lips possessing dentigerous ridges, the presence of interlabia, the ventriculus and ventricular appendage, the excretory pore just posterior to nerve ring and the absence of an intestinal caecum. In *Porrocaecum*, *P. moraveci* sp. n. is similar to the following species in having short interlabia (c. 1/3 length of lips), long intestinal caecum (c. 2/3 length of esophagus) and short spicules (0.60–1.00 mm), including *P. angusticolle* (Molin, 1860),* P. depressum* (Zeder, 1800), *P. circum* Wang, 1965 and *P. phalacrocoracis* Yamaguti, 1941 [2, 19, 20, 22, 36, 37].

*Porrocaecum moraveci* sp. n. differs from *P. phalacrocoracis* and *P. circum* by its distinctly shorter esophagus in both sexes (male 3.61 mm, female 4.68–6.61 mm in the new species vs male 2.08–3.20 mm, female 2.60–3.84 mm in *P. phalacrocoracis* and *P. circum*), unequal spicules (vs spicules equal in length in the latter two species), slightly less number of precloacal papillae (20 pairs vs 21–23 pairs in *P. phalacrocoracis* and *P. circum*) and much smaller body length of female (117.0–138.0 mm in *P. moraveci* sp. n. vs 50.0–65.0 mm in the latter two species).

The new species can be differentiated from *P. angusticolle* by having no cervical alae (vs cervical alae starting at base of subventral lips in *P. angusticolle*) and distinctly unequal spicules (vs spicules almost equal in length in the latter). *Porrocaecum depressum* has been reported from various birds of Accipitriformes, Falconiformes, Strigiformes worldwide, and there are considerable morphological variations in the lengths of the body, esophagus and spicules, the number and arrangement of caudal papillae and the morphology of the tail tip [2, 18–20, 38, 39]. Although the new species is rather similar to *P. depressum*, it is different from *P. depressum* by distinctly unequal spicules (vs spicules almost equal in length in *P. depressum*). Moreover, pairwise comparison of the genetic data of *P. moraveci* with those of *P. angusticolle* and *P. depressum* showed 1.88% (*P. angusticolle*) and 10.8% (*P. depressum*) of nucleotide divergence in the 28S region, 8.30% (*P. angusticolle*) to 14.5% (*P. depressum*) of nucleotide divergence in the ITS region and 7.98–8.18% (*P. angusticolle*) to 10.1% (*P. depressum*) of nucleotide divergence in the *cox*2 region, respectively, which strongly supports the new species being different from *P. angusticolle* and *P. depressum*.

### General characterization of the complete mitogenomes of* Porrocaecum (Porrocaecum) moraveci* sp. n. and* P. (Laymanicaecum) reticulatum*

**Fig. 4:**
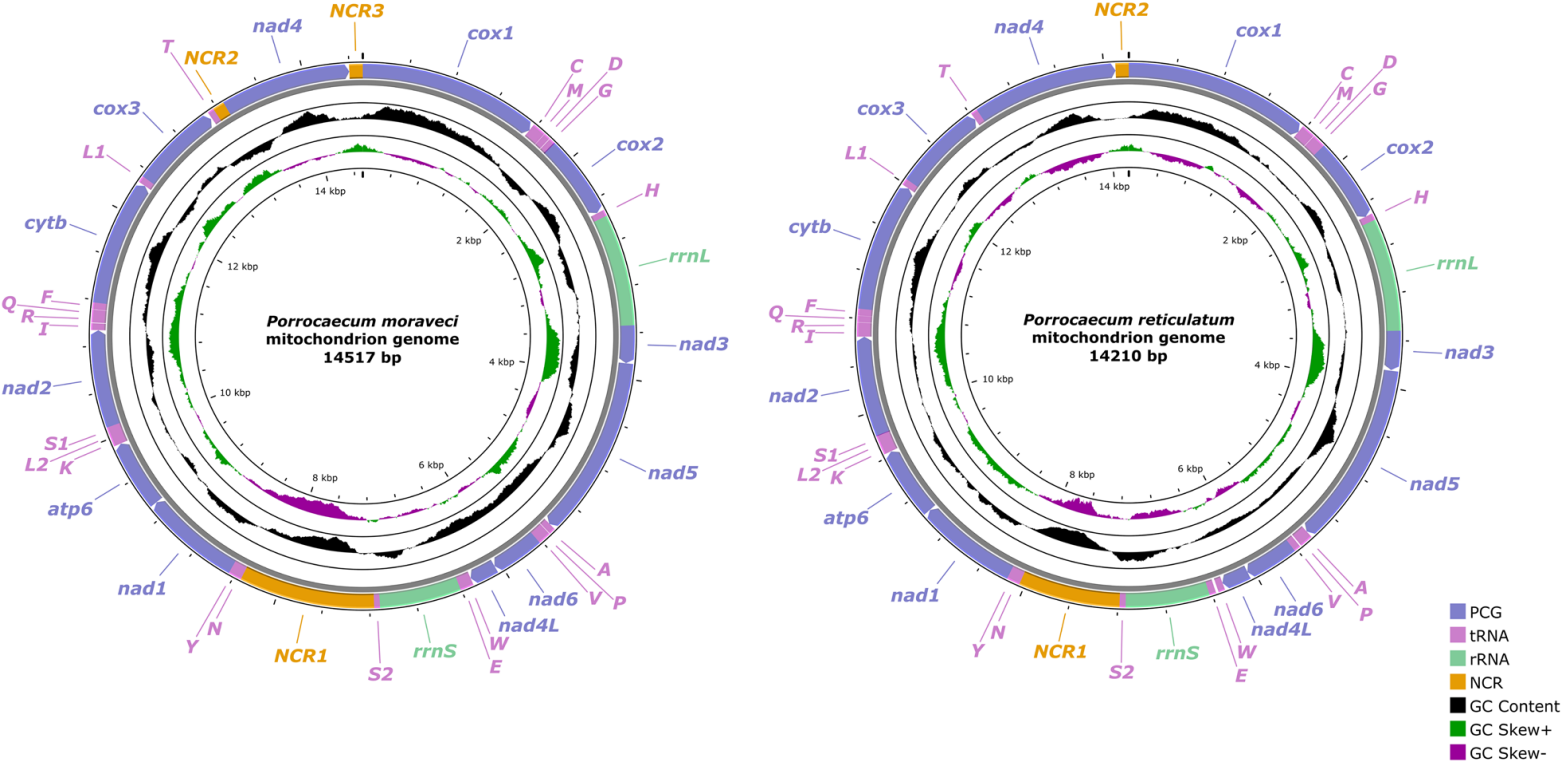
Gene maps of the mitochondrial genomes of *Porrocaecum moraveci* n. sp. and *Porrocaecum reticulatum*. NCR, Non-coding region; PCG, protein-coding gene; rRNA, ribosomal RNA; tRNA, transfer RNA

The circular mitogenomes of *P. moraveci* sp. n. and *P. reticulatum* are 14,517 bp and 14,210 bp in length, respectively, and both contain 36 genes, including 12 PCGs (missing *atp*8) (*cox*1–3, *cyt*b, *nad*1–6, *nad*4L and *atp*6), 22 tRNA genes and two rRNA genes (*rrnL* and *rrnS*) (Fig. 4; Tables 3, 4). There are three non-coding regions in the mitogenome of *P. moraveci* sp. n.: NCR1, which is 1173 bp and located between *tRNA-Ser2* and *tRNA-Asn*; NCR2, which is 101 bp and located between *tRNA-Thr* and *nad*4; and NCR3, which is 117 bp and located between *nad*4 and *cox*1. In comparison, in the mitogenome of *P. reticulatum* there are only two non-coding regions: NCR1, which is 860 bp and located between *tRNA-Ser2* and *tRNA-Asn*; and NCR2, which is 113 bp and located between *nad*4 and *cox*1) (Fig. 4; Tables 3, 4). All genes are transcribed from the same DNA strand. The nucleotide contents of *P. moraveci* sp. n. and *P. reticulatum* mitogenomes are provided in Table 4. The overall A+T content in the mitogenomes of *P. moraveci* sp. n. and *P. reticulatum* is 69.95% and 67.22%, respectively, with both showing a strong nucleotide compositional bias toward A+T (Table 5).

**Table 3:** Annotations and gene organization of *Porrocaecum moraveci* sp. n

Gene	Type	Start (bp)	End (bp)	Length (bp)	Start codon	Stop codon	Anticodon	Strand^a^	Gap or overlap^b^
*cox*1	CDS	1	1575	1575	TTG	TAG		+	− 1
tRNA-Cys ©)	tRNA	1575	1632	58			GCA	+	0
tRNA-Met (M)	tRNA	1633	1693	61			CAU	+	2
tRNA-Asp (D)	tRNA	1696	1752	57			GUC	+	4
tRNA-Gly (G)	tRNA	1757	1812	56			UCC	+	− 9
*cox*2	CDS	1804	2514	711	TTG	TAG		+	2
tRNA-His (H)	tRNA	2517	2571	55			GUG	+	0
*rrnL*	rRNA	2572	3537	966				+	0
*nad*3	CDS	3538	3867	330	GTG	TAG		+	0
*nad*5	CDS	3868	5451	1584	ATT	TAG		+	- 2
tRNA-Ala (A)	tRNA	5450	5505	56			UGC	+	4
tRNA-Pro (P)	tRNA	5510	5565	56			UGG	+	0
tRNA-Val (V)	tRNA	5566	5621	56			UAC	+	0
*nad*6	CDS	5622	6056	435	TTG	TAG		+	0
*nad*4L	CDS	6057	6290	234	ATT	TAG		+	0
tRNA-Trp (W)	tRNA	6291	6348	58			UCA	+	0
tRNA-Glu (E)	tRNA	6349	6407	59			UUC	+	0
*rrnS*	rRNA	6408	7112	705				+	0
tRNA-Ser2 (S2)	tRNA	7113	7162	50			UGA	+	0
NCR1	Non-coding region	7163	8335	1173				+	0
tRNA-Asn (N)	tRNA	8336	8391	56			GUU	+	0
tRNA-Tyr (Y)	tRNA	8392	8450	59			GUA	+	0
*nad*1	CDS	8451	9323	873	TTG	TAG		+	1
*atp*6	CDS	9325	9924	600	ATT	TAA		+	1
tRNA-Lys (K)	tRNA	9926	9988	63			UUU	+	0
tRNA-Leu2 (L2)	tRNA	9989	10,043	55			UAA	+	0
tRNA-Ser1 (S1)	tRNA	10,044	10,096	53			UCU	+	0
*nad*2	CDS	10,097	10,940	844	GTG	T		+	0
tRNA-Ile (I)	tRNA	10,941	10,999	59			GAU	+	5
tRNA-Arg ®)	tRNA	11,005	11,060	56			ACG	+	0
tRNA-Gln (Q)	tRNA	11,061	11,115	55			UUG	+	2
tRNA-Phe (F)	tRNA	11,118	11,176	59			GAA	+	0
*cyt*b	CDS	11,177	12,281	1105	TTG	T		+	0
tRNA-Leu1 (L1)	tRNA	12,282	12,338	57			UAG	+	0
*cox*3	CDS	12,339	13,106	768	TTG	TAG		+	10
tRNA-Thr (T)	tRNA	13,117	13,171	55			UGU	+	0
NCR2	Non-coding region	13,172	13,272	101				+	0
*nad*4	CDS	13,273	14,400	1128	TTG	TAA		+	0
NCR3	Non-coding region	14,401	14,517	117				+	

*CDS* Coding sequence (coding region of a gene),* NCR* non-coding region, * tRNA* transfer RNA

^a^The forward strand is marked as “+” and the reverse strand is marked as “−”

^b^Positive number in the “Gap or overlap” column indicates the length of the intergenic sequence, with negative numbers indicating the length (absolute number) that adjacent genes overlap (negative sign)

**Table 4:** Annotations and gene organization of *Porrocaecum reticulatum*

Gene	Type	Start (bp)	End (bp)	Length (bp)	Start codon	Stop codon	Anticodon	Strand^a^	Gap or overlap^b^
*cox*1	CDS	1	1575	1575	TTG	TAG		+	− 1
tRNA-Cys©)	tRNA	1575	1631	57			GCA	+	0
tRNA-Met(M)	tRNA	1632	1693	62			CAU	+	3
tRNA-Asp(D)	tRNA	1697	1753	57			GUC	+	0
tRNA-Gly(G)	tRNA	1754	1810	57			UCC	+	0
*cox*2	CDS	1811	2506	696	TTG	TAG		+	− 2
tRNA-His(H)	tRNA	2505	2562	58			GUG	+	0
*rrn*L	rRNA	2563	3515	953				+	0
*nad*3	CDS	3516	3851	336	GTG	TAG		+	6
*nad*5	CDS	3858	5439	1582	ATT	T		+	0
tRNA-Ala(A)	tRNA	5440	5495	56			UGC	+	0
tRNA-Pro(P)	tRNA	5496	5552	57			UGG	+	11
tRNA-Val(V)	tRNA	5564	5620	57			UAC	+	0
*nad*6	CDS	5621	6055	435	TTG	TAA		+	− 1
*nad*4L	CDS	6055	6288	234	ATT	TAA		+	− 1
tRNA-Trp(W)	tRNA	6288	6344	57			UCA	+	22
tRNA-Glu(E)	tRNA	6367	6423	57			UUC	+	0
*rrn*S	rRNA	6424	7132	709				+	0
tRNA-Ser2(S2)	tRNA	7133	7184	52			UGA	+	0
NCR1	Non-coding region	7185	8044	860				+	0
tRNA-Asn(N)	tRNA	8045	8101	57			GUU	+	0
tRNA-Tyr(Y)	tRNA	8102	8156	55			GUA	+	0
*nad*1	CDS	8157	9029	873	TTG	TAA		+	2
*atp*6	CDS	9032	9631	600	ATT	TAA		+	2
tRNA-Lys(K)	tRNA	9634	9695	62			UUU	+	0
tRNA-Leu2(L2)	tRNA	9696	9750	55			UAA	+	0
tRNA-Ser1(S1)	tRNA	9751	9802	52			UCU	+	− 6
*nad*2	CDS	9797	10,646	850	TTG	T		+	0
tRNA-Ile(I)	tRNA	10,647	10,708	62			GAU	+	0
tRNA-Arg®)	tRNA	10,709	10,764	56			ACG	+	2
tRNA-Gln(Q)	tRNA	10,767	10,821	55			UUG	+	1
tRNA-Phe(F)	tRNA	10,823	10,879	57			GAA	+	0
*cyt*b	CDS	10,880	11,986	1107	TTG	TAG		+	− 1
tRNA-Leu1(L1)	tRNA	11,986	12,041	56			UAG	+	0
*cox*3	CDS	12,042	12,809	768	ATT	TAG		+	1
tRNA-Thr(T)	tRNA	12,811	12,867	57			UGU	+	0
*nad*4	CDS	12,868	14,097	1230	TTG	TAG		+	0
NCR2	Non-coding region	14,098	14,210	113				+	

*CDS* Coding sequence (coding region of a gene),* NCR* non-coding region, * tRNA* transfer RNA

^a^The forward strand is marked as “+” and the reverse strand is marked as “−”

^b^Positive number in the “Gap or overlap” column indicates the length of the intergenic sequence, with negative numbers indicating the length (absolute number) that adjacent genes overlap (negative sign)

**Table 5:** Base composition and skewness of *Porrocaecum moraveci* sp. n. and *P. reticulatum*

Location/species	Base composition					Skewness		Total (bp)
	A (%)	T (%)	C (%)	G (%)	A+T content (%)	AT skew	GC skew	
*Porrocaecum moraveci sp. n*								
Whole mitochondrial genome	21.99	47.95	8.27	21.78	69.95	− 0.37	0.45	14,517
PCGs	18.56	50.50	8.04	22.90	69.06	− 0.46	0.48	10,185
Condon position								
− 1st codon	26.58	40.80	8.21	24.40	67.38	− 0.21	0.50	3395
- 2nd codon	18.76	51.25	13.52	16.47	70.01	− 0.46	0.10	3395
- 3rd codon	10.34	59.44	2.39	27.84	69.78	− 0.70	0.84	3395
tRNAs	29.38	41.15	8.65	20.82	70.54	− 0.17	0.41	1249
rRNAs	26.93	43.51	8.26	21.30	70.44	− 0.24	0.44	1671
- *rrn*L	24.95	46.69	7.45	20.91	71.64	− 0.30	0.47	966
*- rrn*S	29.65	39.15	9.36	21.84	68.79	− 0.14	0.40	705
NCR 1	36.57	38.62	10.66	14.15	75.19	− 0.03	0.14	1173
NCR 2	17.82	61.38	4.95	15.84	79.21	− 0.55	0.52	101
NCR 3	29.91	43.59	5.13	21.37	73.50	− 0.19	0.61	117
*Porrocaecum reticulatum*								
Whole mitochondrial genome	20.70	46.52	9.25	23.34	67.22	− 0.38	0.43	14,210
PCGs	17.62	48.88	9.31	24.19	66.50	− 0.47	0.44	10,284
Condon position								
− 1st codon	25.77	40.29	9.50	24.43	66.06	− 0.22	0.44	3428
− 2nd codon	18.38	50.90	13.86	16.86	69.28	− 0.47	0.10	3428
− 3rd codon	8.69	55.46	4.58	31.27	64.15	− 0.73	0.75	3428
tRNAs	26.78	39.33	10.23	23.66	66.11	− 0.19	0.40	1251
rRNAs	25.63	41.76	9.93	22.68	67.39	− 0.24	0.39	1662
*- rrn*L	23.92	45.54	8.39	22.14	69.46	− 0.31	0.45	953
*- rrn*S	27.93	36.67	11.99	23.41	64.60	− 0.14	0.32	709
NCR 1	37.67	39.30	6.86	13.14	76.98	− 0.02	0.31	860
NCR 2	30.97	43.36	4.42	21.24	74.34	− 0.17	0.66	113

*NCR* Non-coding region, *PCG* Protein-coding gene, rRNA ribosomal RNA,* tRNA* transfer RNA

**Fig. 5:**
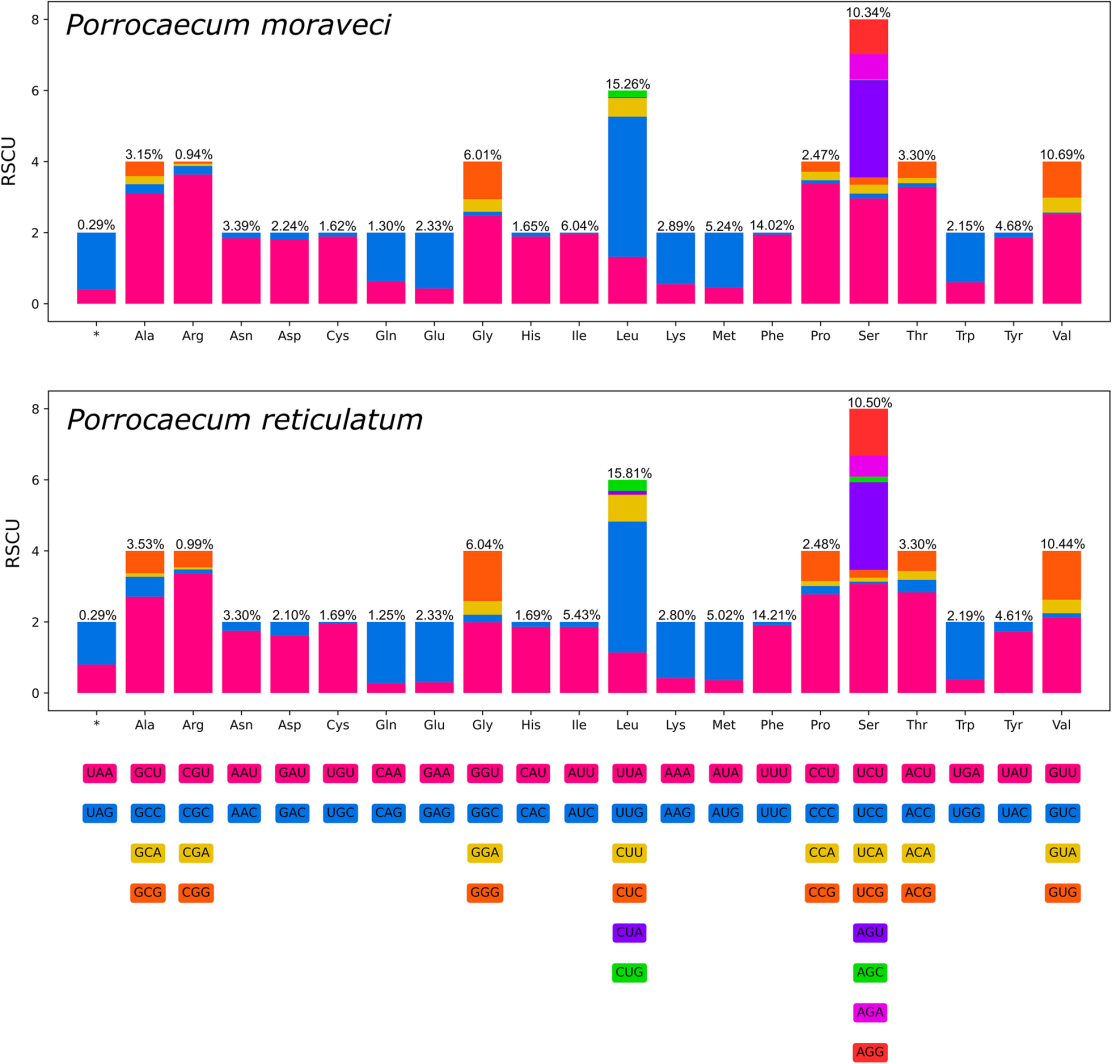
RSCU of *Porrocaecum moraveci* n. sp. and *P. reticulatum*. Codon families (in alphabetical order, from left to right) are provided below the horizontal axis. Values at the top of each bar represent amino acid usage in percentage. RSCU, Relative synonymous codon usage

The 12 PCGs of the mitogenomes of *P. moraveci* sp. n. and *P. reticulatum* are 10,185 bp and 10,284 bp in length (excluding termination codons) and ranged in size from 234 bp (*nad4L*) to 1584 bp (*nad*5), which encoded 3395 and 3428 amino acids, respectively (Tables 3–5). Among the 12 PCGs of *P. moraveci* sp. n., seven genes (*cox*1*, cox*2*, cox*3*, cyt*b*, nad*1*, nad*4 and *nad*6) used TTG as the start codon, whereas three genes (*nad*5, *nad*4L and *atp*6) used ATT; GTG was used as the start codon by the *nad*2 and *nad*3 genes. TAG was the most commonly used termination codon (*cox*1, *cox*2, *cox*3, *nad*1, *nad*3, *nad*4L, *nad*5 and *nad*6); two genes (*atp*6 and *nad*4) used TAA, and the incomplete termination codon T was inferred for the *nad*2 and *cyt*b genes (Table 3). Among the 12 PCGs of *P. reticulatum*, seven genes (*cox*1, *cox*2, *cyt*b, *nad*1, *nad*2, *nad*4 and *nad*6) used TTG as the start codon, whereas four genes (*atp*6*, nad*4L*, nad*5 and *cox*3) used ATT; GTG was used as the start codon by only the *nad*3 gene. TAG was the most commonly used termination codon (*cox*1, *cox*2, *cox*3, *nad*3, *nad*4 and *cyt*b); four genes (*nad*1, *nad*4L, *nad*6 and *atp*6) used TAA, and the incomplete termination codon T was inferred only for the *nad*2 and *nad*5 genes (Table 4). The components and usages of codons in the mitogenomes of *P. moraveci* sp. n. and *P. reticulatum* are shown in Fig. 5 and in Tables 3, 4.

**Fig. 6:**
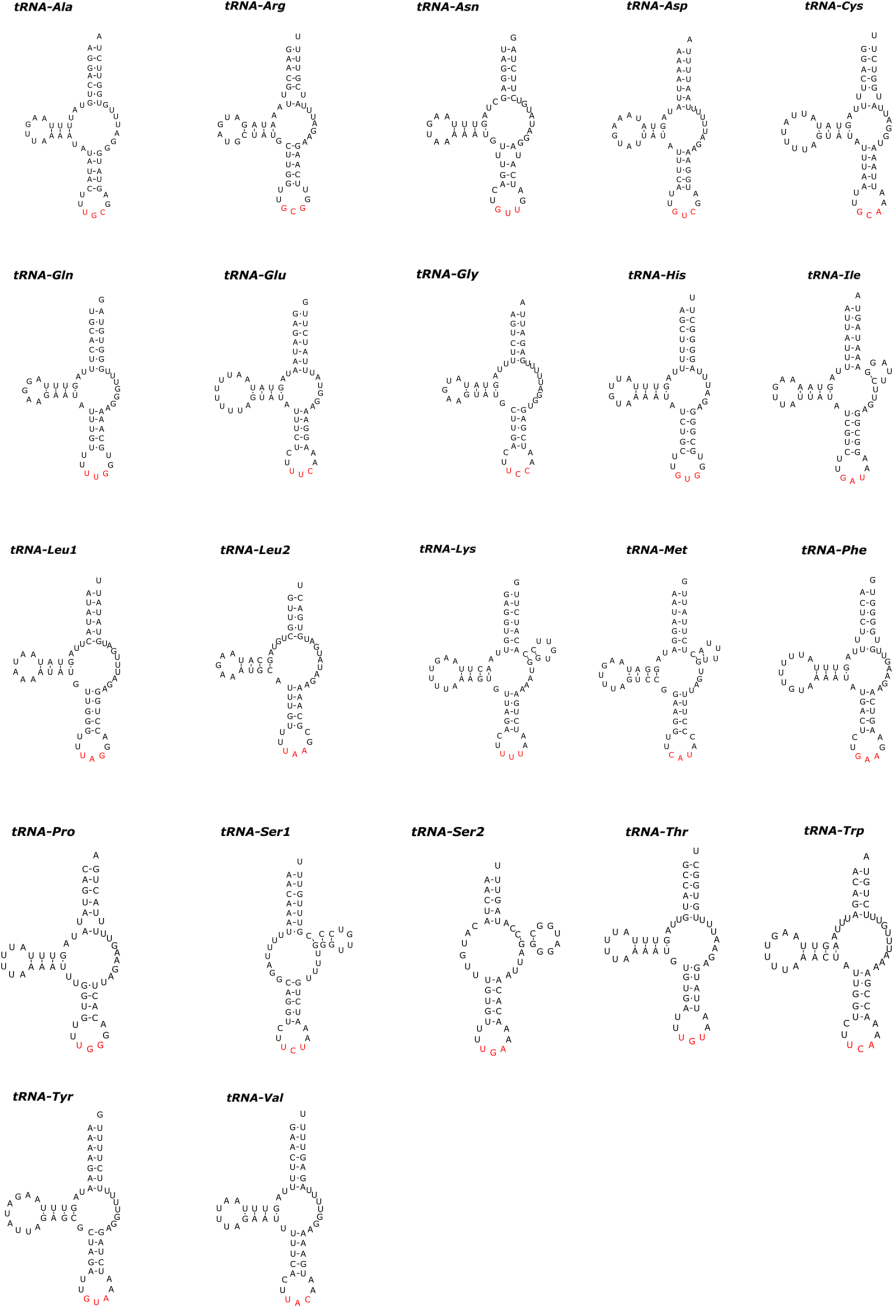
Inferred secondary structures of 22 tRNAs in the mitogenome of *Porrocaecum moraveci* n. sp. Lines between bases indicate Watson–Crick bonds, dots indicate GU bonds and bases in red represent anticodons. tRNA, Transfer RNA

**Fig. 7:**
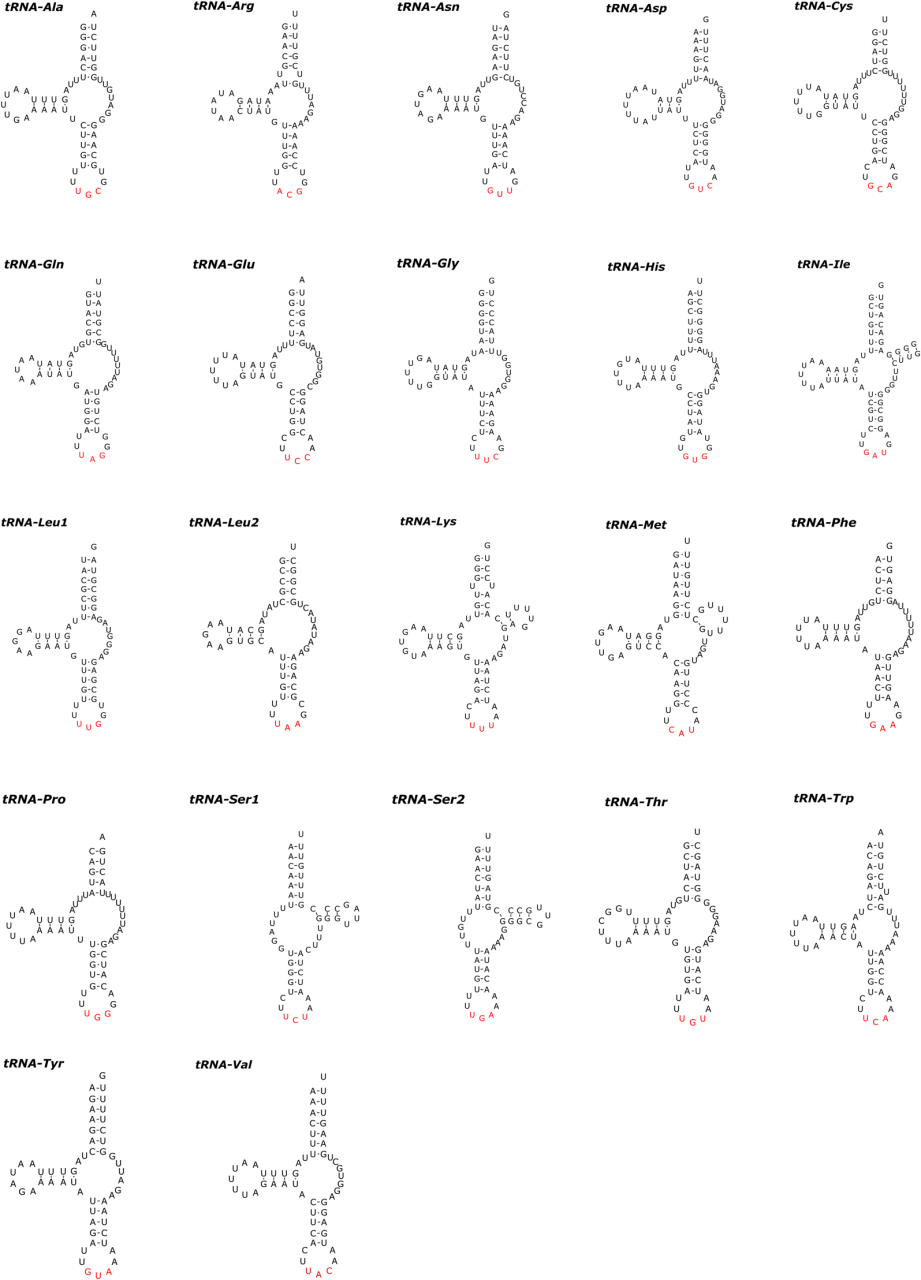
Inferred secondary structures of 22 tRNAs in the mitogenome of *Porrocaecum reticulatum*. Lines between bases indicate Watson–Crick bonds, dots indicate GU bonds and bases in red represent anticodons. tRNA, Transfer RNA

In the mitogenomes of *P. moraveci* sp. n. and *P. reticulatum*, 22 tRNAs were identified. The length of these 22 tRNAs and their anticodon secondary structures are shown in Tables 3 and 4 and in Figs. 6 and 7. Two rRNAs (*rrnL* located between *tRNA-His* and *nad*3, and *rrnS* located between *tRNA-Glu* and *tRNA-Ser*2) were identified in the mitogenomes of *P. moraveci* sp. n. and *P. reticulatum* (Fig. 4); the length of each gene is provided in Tables 3 and 4.

The gene arrangement of 36 genes in the mitogenomes of *P. moraveci* sp. n. and *P. reticulatum* are both in the following order: *cox*1, *tRNA-Cys*, *tRNA-Met*, *tRNA-Asp*, *tRNA-Gly*, *cox*2, *tRNA-His*, *rrnL*, *nad*3, *nad*5, *tRNA-Ala*, *tRNA-Pro*, *tRNA-Val*, *nad*6, *nad*4l, *tRNA-Trp*, *tRNA-Glu*, *rrnS*, *tRNA-Ser2*, *tRNA-Asn*, *tRNA-Tyr*, *nad*1, *atp*6, *tRNA-Lys*, *tRNA-Leu2*, *tRNA-Ser1*, *nad*2, *tRNA-Ile*, *tRNA-Arg*, *tRNA-Gln*, *tRNA-Phe*, *cyt*b, *tRNA-Leu*1, *cox*3, *tRNA-Thr*, *nad*4 (Fig. 4). This follows the GA3 type of gene arrangement.

### Phylogenetic analyses

**Fig. 8:**
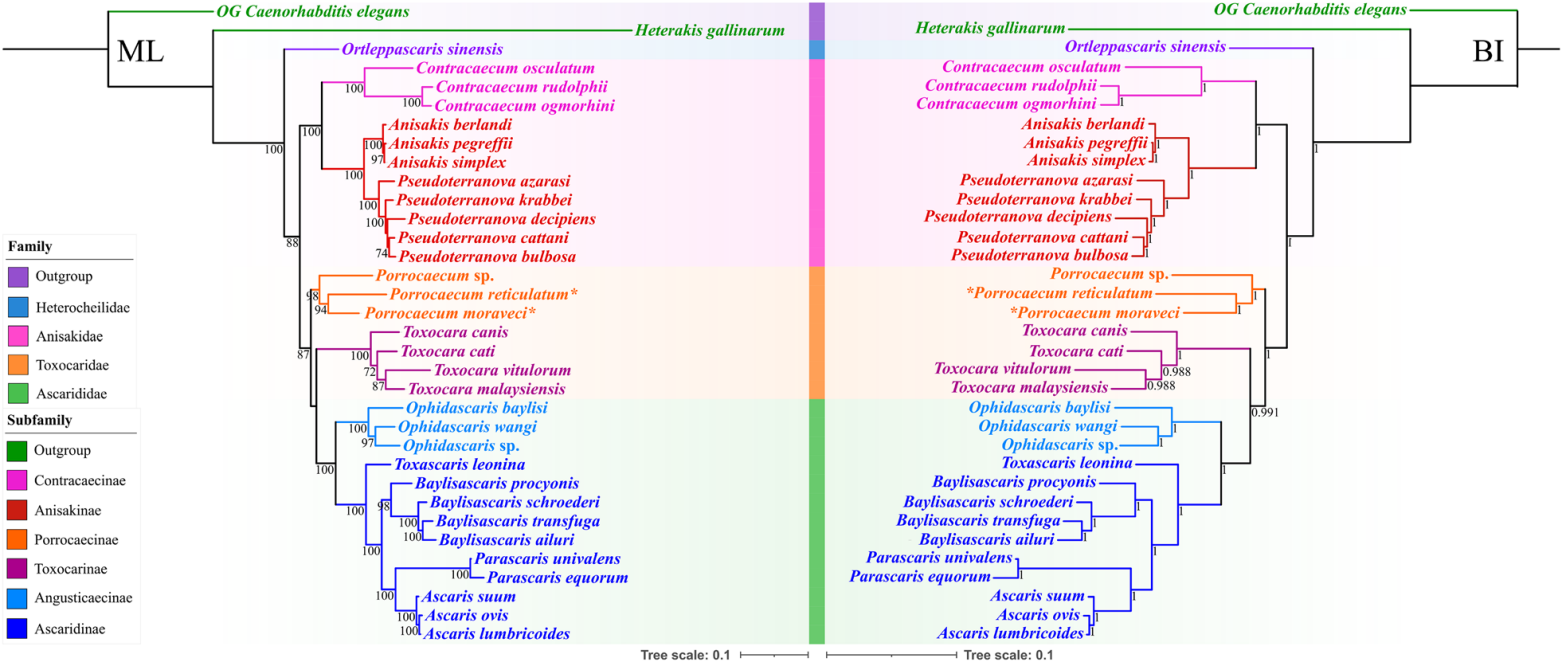
Phylogenetic relationships among ascaridoid nematodes inferred from ML and BI methods based on the amino acid sequences of 12 PCGs of mitochondrial genomes. *Caenorhabditis elegans* (Rhabditida: Rhabditoidea) and *Heterakis gallinarum* (Ascaridomorph: Heterakoidea) were chosen as the outgroup. Bootstrap values ≥ 70 and Bayesian posterior probabilities values ≥ 0.70 are shown in the phylogenetic trees. Asterisk indicates *Porrocaecum moraveci* n. sp. and *P. reticulatum*. BI, Bayesian inference; ML, maximum likelihood; PCGs, protein-coding genes

The phylogenetic trees of ascaridoid nematodes constructed using the BI and ML methods based on the amino acid sequences of 12 PCGs of mitogenomes were found to have similar topologies, and both showed that the family Heterocheilidae (including only *Ortleppascaris sinensis*) is at the base of the phylogenetic trees, which formed a sister clade to the remaining Ascaridoidea (Fig. 8). The representatives of the family Anisakidae were divided into two subclades, representing the subfamilies Contracaecinae (including *Contracaecum* spp.) and Anisakinae (including *Anisakis* spp. and *Pseudoterranova* spp.), respectively. Phylogenetic analyses did not support the monophyly of the family Toxocaridae (including *Toxocara* spp. and *Porrocaecum* spp.), which showed the representatives of *Toxocara* clustered together with species of the family Ascarididae (including *Ophidascaris* spp., *Toxascaris leonina*, *Baylisascaris* spp., *Parascaris* spp. and *Ascaris* spp.), with strong support in the BI tree, but weak support in the ML tree (Fig. 8). In the genus *Porrocaecum*, both of the phylogenetic results showed that *P. moraveci* sp. n. is a sister to *P. reticulatum* with strong support (Fig. 8).

**Fig. 9:**
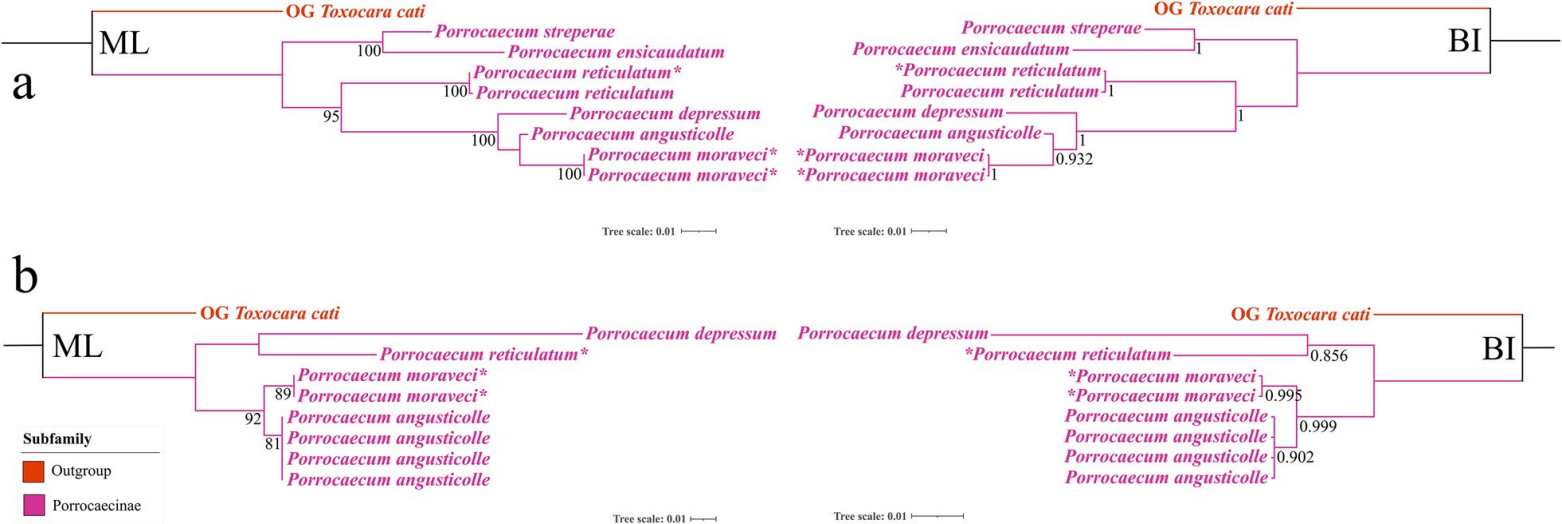
Phylogenetic relationships among *Porrocaecum* species inferred from ML and BI methods. *Toxocara cati* (Ascaridoidea: Toxocaridae) was chosen as the outgroup. **a** Phylogenetic trees constructed using 18S + ITS sequence data, **b** phylogenetic trees constructed using 28S sequence data. Bootstrap values ≥ 70 and Bayesian posterior probabilities values ≥ 0.70 are shown in the phylogenetic trees. Asterisk indicates *Porrocaecum moraveci* n. sp. and *P. reticulatum*. BI, Bayesian inference; ML, maximum likelihood; OG, outgroup

In the phylogenetic trees of *Porrocaecum* species constructed using BI and ML methods based on the 18S + ITS and 28S sequence data, *P. moraveci* sp. n. both showed a sister relationship with *P. angusticolle* with strong support. In the phylogenetic trees based on the 18S + ITS sequence data, *P. reticulatum* was clustered with *P. depressum* + *P. moraveci* sp. n. + *P. angusticolle*; but *P. reticulatum* was sister to *P. depressum* in the phylogenetic trees based on the 28S sequence data (Fig. 9).

## Discussion

The mitogenomes are very useful for understanding the epidemiology, population genetics and molecular phylogeny of ascaridoid nematodes. However, there are sequenced mitogenomes for only 30 species of ascaridoids (Table 1). In the genus *Porrocaecum*, only one unidentified species, *Porrocaecum* sp., has been genetically sequenced for the mitogenome. In the study reported here, we sequenced and assembled the complete mitogenomes of *P. moraveci* sp. n. and *P. reticulatum* for the first time.

The complete mitogenomes of *P. moraveci* sp. n. and *P. reticulatum* are 14,517 bp and 14,210 bp in length, respectively; as such, their lengths are similar to that of *Porrocaecum* sp. (14,568 bp) and *Toxocara* spp. (14,029–15045 bp) [14, 17, 40]. The lack of *atp*8 in the mitogenomes of *P. moraveci* sp. n. and *P. reticulatum* is typical of most of the available mitogenomes of nematodes, with the exception of *Trichinella* spp. and *Trichuris* spp., both of which have the *atp*8 gene [41–46]. The gene arrangement of the mitogenomes of *P. moraveci* sp. n. and *P. reticulatum* both belong to the GA3 type, agreeing well with that of *Porrocaecum* sp. and the other ascaridoid species [14, 17, 40, 47–50]. The overall A + T contents in the mitogenomes of *P. moraveci* sp. n. (69.95%) and *P. reticulatum* (67.22%) are distinctly lower than that of *Porrocaecum* sp. (71.42%). In fact, the overall A + T contents of *P. reticulatum* is the lowest of all available mitogenomes of ascaridoid nematodes. Additionally, comparative mitogenomics revealed that *P. reticulatum* and *Porrocaecum* sp. both have two non-coding regions in their mitogenomes, while there are three non-coding regions in the mitogenome of *P. moraveci* sp. n., which is different from all of the ascaridoid mitogenomes reported so far.

Although some recent phylogenies based on molecular studies have improved and challenged the traditional classification of the superfamily Ascaridoidea [7, 14–17, 51], phylogenetic relationships within several lineages of the Ascaridoidea remain unresolved due to a paucity of genetic data. In 1974, Hartwich erected the family Toxocaridae [4], but he subsequently degraded it as a subfamily in the Ascarididae, a change that was widely accepted in subsequent studies [8, 9, 52]. In his 1974 classification, Hartwich listed three genera in the subfamily Toxocarinae, including *Toxocara*, *Porrocaecum* and *Paradujardinia* [4]. Later, Sprent (in 1983) transferred *Paradujardinia* into the family Heterocheilidae, a change that was supported by Gibson (in 1983) and Fagerholm (in 1991) [8, 9, 53]. In 1958,Osche considered that the family Toxocaridae was valid and erected a new subfamily Porrocaecinae for the genus *Porrocaecum* in the Toxocaridae [54]. However, Osche’s proposal has received little attention since its inception, and only Chabaud (in 1965) suggested treating the Porrocaecinae as a tribe Porrocaecinea [55]. Our phylogenetic analyses of ascaridoids based on the amino acid sequences of 12 PCGs using ML and BI inference showed that *Porrocaecum* and *Toxocara* have no close relationship and that the Toxocaridae/Toxocarinae classification proposed by Hartwich is not a monophyletic group; these findings conflict with these above-mentioned classifications but are roughly consistent with some previous molecular phylogenetics results [14, 16, 17, 56].

Mozgovoi [2] erected the subgenus *Laymanicaecum* in *Porrocaecum* based on the criterion of presence of gubernaculum in the male, and two species *P.* (*Laymanicaecum*) *laymani* Mozgovoi, 1950 and *P.* (*Laymanicaecum*) *reticulatum* (Mozgovoi, 1953) were assigned to the subgenus *Laymanicaecum*. However, *P.* (*Laymanicaecum*) *laymani* was subsequently transferred into the genus *Mawsonascaris* [57]; thus, *P.* (*Laymanicaecum*) *reticulatum* is the only species with gubernaculum in the male in *Porrocaecum*. In fact, as an important generic criterion, the gubernaculum is most often absent in the Anisakidae, Ascarididae, Toxocaridae and Raphidascarididae. Consequently, the systematic status of *P. reticulatum* and the subgenus *Laymanicaecum* in the Ascaridoidea is very puzzling. The present molecular phylogenetic analyses based on the 18S + ITS, 28S sequence data and 12 PCGs all showed that *P. (Laymanicaecum) reticulatum* nested in the representatives of the subgenus *Porrocaecum*, which supports the invalidity of the classification of *Laymanicaecum* as a subgenus and also indicates that care should be taken when using the gubernaculum as an important morphological character for delimitation of some genera within the Ascaridoidea.

Towards the integration of the present phylogenetic results and the traditional classification, we propose (i) to resurrect the subfamily Porrocaecinae including only the genus *Porrocaecum*; and (ii), and to degrade the Toxocaridae as a subfamily of the Ascarididae including only the genus *Toxocara*. Consequently, the Ascarididae should include four subfamilies, namely Ascaridinae, Porrocaecinae, Toxocaridae and Angusticaecinae. The present phylogenetic study represents a substantial step toward clarifying the evolutionary relationships of the subfamilies and families in the Ascaridoidea. However, we do not propose making any immediate systematic changes in the Ascaridoidea because a more rigorous study with broader representation of the Ascarididae and Ascaridoidea is required.

## Conclusions

A new species of *Porrocaecum*, *P. moraveci* n. sp., was described based on the integration of morphological and genetic evidence from specimens collected from *C. aeruginosus* in the Czech Republic. The genetic characterization of the complete mitochondrial genomes of *P. moraveci* n. sp. and *P. reticulatum* was reported for the first time. Comparative mitogenomics revealed that *P. moraveci* n. sp. represents the first species with three non-coding regions and *P. reticulatum* has the lowest overall A + T content in the available mitogenomes of ascaridoid nematodes reported so far. Our phylogenetic results challenge the monophyly of the Toxocaridae and show that *Porrocaecum* and *Toxocara* do not have an affinity. The mitogenomic phylogenies determined here suggest (i) to degrade the Toxocaridae as a subfamily of the Ascarididae including only the genus *Toxocara*; and (ii) to resurrect the subfamily Porrocaecinae including only the genus *Porrocaecum*. The validity of the subgenus *Laymanicaecum* in *Porrocaecum* was also rejected. The present study enriches the database of ascaridoid mitogenomes and provides a new insight into the systematics of the superfamily Ascaridoidea.

### Supplementary Information

**Additional file 1:** **Table S1.** The partitioning schemes and the optimal model selected for each combination of partition for the BI inference.

## Data Availability

The nuclear and mitochondrial DNA sequences of *Porrocaecum moraveci* n. sp. and *P. reticulatum* obtained in the present study were deposited in GenBank database (sequences of *Porrocaecum moraveci* n. sp. under the accession numbers: OQ858491, OQ858492 (18S), OQ858562, OQ858563 (28S), OQ858560, OQ858561 (ITS), OQ863051 (mitochondrial genome); sequences of *P. reticulatum* under the accession numbers: OQ851895 (18S), OQ863745 (28S), OQ857284 (ITS), OQ863050 (mitochondrial genome). Type specimens of *Porrocaecum moraveci* n. sp. were deposited in the College of Life Sciences, Hebei Normal University, Hebei Province, China (under the accession numbers R: HBNU–N–B20220021GL, HBNU–N–B20220022GL and HBNU–N–B20220023GL).

## Abbreviations

BI: Bayesian inference
BIC: Bayesian information criterion
ITS: Internal transcribed spacer
ML: Maximum likelihood
mt: Mitochondrial
PCG: Protein-coding gene
SEM: Scanning electron microscopy
18S: Small ribosomal subunit
28S: Large ribosomal subunit

## References

[1] Skrjabin KI, Schikhobalova NP, Mozgovoi AA. Key to parasitic nematodes. Volume II: Oxyurata and Ascaridata. Moscow: Izdatei'stvo Akademii Nauk SSSR; 1951.

[2] Mozgovoi AA. Ascaridata of animals and man and the diseases caused by them. Volume 2: Part 1 of Osnovy nematodologii. Moscow: Izdatel’stvo Akademii Nauk SSSR; 1953.

[3] Yamaguti S. Systema helminthum. The nematodes of vertebrates, vol. 3. New York: Interscience; 1961.

[4] Hartwich G, Anderson RC, Chabaud AG, Willmott S (1974). Keys to genera of the Ascaridoidea. CIH keys to the nematode parasites of vertebrates.

[5] Bruce NL, Adlard RD, Cannon LRG (1994). Synoptic checklist of ascaridoid parasites (Nematoda) from fish hosts. Invertebr Taxon.

[6] Hodda M, Zhang ZQ (2011). Phylum Nematoda Cobb 1932. Animal biodiversity: an outline of higher-level classification and survey of taxonomic richness (Addenda 2013).

[7] Li L, Lü L, Nadler SA, Gibson DI, Zhang LP, Chen HX (2018). Molecular phylogeny and dating reveal a terrestrial origin in the early carboniferous for ascaridoid nematodes. Syst Biol.

[8] Gibson DI. The systematics of ascaridoid nematodes—a current assessment. In: Stone R, Platt HM, Khalil LF, editors. Concepts in nematode systematics. Systematics Association special volume 22. London: Academic Press; 1983. p. 321–38.

[9] Fagerholm HP (1991). Systematic implications of male caudal morphology in ascaridoid nematode parasites. Syst Parasitol.

[10] Gibbons LM, Jacobs DE, Sani RA (2001). *Toxocara malaysiensis* n. sp. (Nematoda: Ascaridoidea) from the domestic cat (*Felis catus* Linnaeus, 1758). J Parasitol.

[11] Anderson RC (2000). Nematode parasites of vertebrates their development and transmission.

[12] Macpherson CN (2013). The epidemiology and public health importance of toxocariasis: a zoonosis of global importance. Int J Parasitol.

[13] Ma GX, Holland CV, Wang T, Hofmann A, Fan CK, Maizels RM (2017). Human toxocariasis. Lancet Infect Dis.

[14] Han L, Yang YL, Li HM, Zhou XY, Zhou MC, Liu TL (2022). Gene rearrangements in the mitochondrial genome of ten ascaris species and phylogenetic implications for Ascaridoidea and Heterakoidea families. Int J Biol Macromol.

[15] Nadler SA, Hudspeth DS (1998). Ribosomal DNA and phylogeny of the Ascaridoidea (Nemata: Secernentea): implications for morphological evolution and classification. Mol Phylogenet Evol.

[16] Nadler SA, Hudspeth DS (2000). Phylogeny of the Ascaridoidea (Nematoda: Ascaridida) based on three genes and morphology: hypotheses of structural and sequence evolution. J Parasitol.

[17] Xie Y, Wang LD, Chen YJ, Wang Z, Zhu PC, Hu Z (2022). The complete mitogenome of *Toxocara vitulorum*: novel in-sights into the phylogenetics in Toxocaridae. Animals.

[18] Cram E (1927). Bird parasites of nematode suborders Strongylata, Ascaridata and Spirurata. Bull US Nat Mus.

[19] Hartwich G (1959). Revision der vogelparasitischen Nematoden Mitteleuropas I. Die Gattung *Porrocaecum* Railliet and Henry, 1912 (Ascaridoidea). Mitt Zool Mus Berlin.

[20] Fagerholm HP, Overstreet RM, Atkinson CT, Thomas NJ, Hunter DB (2009). Ascaridoid Nematodes: *Contracaecum*, *Porrocaecum*, and *Baylisascaris*. Parasitic diseases of wild birds.

[21] Li L, Guo YN, Zhang LP (2015). *Porrocaecum parvum* n. sp. and *P. reticulatum* (Linstow, 1899) (Nematoda: Ascaridoidea) from birds in China. Syst Parasitol.

[22] Guo N, Sitko J, Chen HX, Li L (2021). Morphological and genetic characterization of *Porrocaecum angusticolle* (Molin, 1860) (Nematoda: Ascaridomorpha) from the common buzzard *Buteo buteo* (Linnaeus) (Accipitriformes: Accipitridae) in Czech Republic. Parasitol Int.

[23] Floyd RM, Rogers AD, Lambshead PJD, Smith CR (2005). Nematode-specific PCR primers for the 18S small subunit rRNA gene. Mol Ecol Notes.

[24] Zhu XQ, D'Amelio S, Paggi L, Gasser RB (2000). Assessing sequence variation in the internal transcribed spacers of ribosomal DNA within and among members of the *Contracaecum osculatum* complex (Nematoda: Ascaridoidea: Anisakidae). Parasitol Res.

[25] Jin JJ, Yu WB, Yang JB, Song Y, De Pamphilis CW, Yi TS (2020). GetOrganelle: a fast and versatile toolkit for accurate de novo assembly of organelle genomes. Genome Biol.

[26] Meng GL, Li YY, Yang CT, Liu SL (2019). MitoZ: A toolkit for animal mitochondrial genome assembly, annotation and visualization. Nucleic Acids Res.

[27] Gruber AR, Bernhart SH, Lorenz R, Picardi E (2015). The viennaRNA web services. RNA bioinformatics, methods in molecular biology.

[28] Bernt M, Merkle D, Ramsch K, Fritzsch G, Perseke M, Bernhard D (2007). Inferring genomic rearrangements based on common intervals. Bioinformatics.

[29] Reuter JS, Mathews DH (2010). RNAstructure: software for RNA secondary structure prediction and analysis. BMC Bioinform.

[30] Lee BD (2018). Python implementation of codon adaptation index. JOSS.

[31] Katoh K, Standley DM (2013). Mafft multiple sequence alignment software version 7: improvements in performance and usability. Mol Biol Evol.

[32] Criscuolo A, Gribaldo S (2010). BMGE (block mapping and gathering with entropy): a new software for selection of phylogenetic informative regions from multiple sequence alignments. BMC Evol Biol.

[33] Zhang D, Gao F, Jakovlić I, Zou H, Zhang J, Li WX (2020). PhyloSuite: An integrated and scalable desktop platform for streamlined molecular sequence data management and evolutionary phylogenetics studies. Mol Ecol Resour.

[34] Minh BQ, Hahn MW, Lanfear R (2020). New methods to calculate concordance factors for phylogenomic datasets. Mol Biol Evol.

[35] Ronquist F, Teslenko M, Van Der Mark P, Ayres DL, Darling A, Höhna S (2012). MrBayes 3.2: efficient Bayesian phylogenetic inference and model choice across a large model space. Syst Biol.

[36] Wang PQ (1965). Notes on some nematodes of the suborder Ascaridata from Fukien, China. Acta Parasitol Sin.

[37] Yamaguti Y (1941). Studies on the helminth fauna of Japan Part 36 Avian nematodes, II. Jpn J Zool..

[38] Yoshino T, Yanai T, Asano M (2012). First record of Porrocaecum depressum (Nematoda: Ascaridoidea), *Craspedorrhynchus* sp. and *Degeeriella* sp. (Insecta: Psocodea) obtained from a Hodgson's hawk eagle, *Spizaetus*
*nipalensis*, in Gifu Prefecture, Japan. Biogeography.

[39] Morgan BB, Schiller EL (1950). *Porrocaecum angusticolle* (Nematoda) in North American hawks. Trans Am Microsc Soc.

[40] Li MW, Lin RQ, Song HQ, Wu XY, Zhu XQ (2008). The complete mitochondrial genomes for three *Toxocara* species of human and animal health significance. BMC Genomics.

[41] Lavrov DV, Brown WM (2001). *Trichinella spiralis* mtDNA: a nematode mitochondrial genome that encodes a putative ATP8 and normally structured tRNAs and has a gene arrangement relatable to those of coelomate metazoans. Genetics.

[42] Liu GH, Gasser RB, Su A, Nejsum P, Peng L, Lin RQ (2012). Clear genetic distinctiveness between human- and pig-derived *Trichuris* based on analyses of mitochondrial datasets. PLoS Negl Trop Dis.

[43] Liu GH, Wang Y, Xu MJ, Zhou DH, Ye YG, Li JY (2012). Characterization of the complete mitochondrial genomes of two whipworms *Trichuris ovis* and *Trichuris discolor* (Nematoda: Trichuridae). Infect Genet Evol.

[44] Liu GH, Gasser RB, Nejsum P, Wang Y, Chen Q, Song HQ (2013). Mitochondrial and nuclear ribosomal DNA evidence supports the existence of a new *Trichuris* species in the endangered François' leaf-monkey. PLoS ONE.

[45] Mohandas N, Jabbar A, Podolska M, Zhu XQ, Littlewood DT, Jex AR (2014). Mitochondrial genomes of Anisakis simplex and Contracaecum osculatum (sensu stricto)–comparisons with selected nematodes. Infect Genet Evol.

[46] Hawash MB, Andersen LO, Gasser RB, Stensvold CR, Nejsum P (2015). Mitochondrial genome analyses suggest multiple *Trichuris* species in humans, baboons, and pigs from different geographical regions. PLoS Negl Trop Dis.

[47] Yamada A, Ikeda N, Ono H (2017). The complete mitochondrial genome of *Anisakis pegreffii* Campana-Rouget and Biocca, 1955, (Nematoda, Chromadorea, Rhabditida, Anisakidae)—clarification of mitogenome sequences of the *Anisakis simplex* species complex. Mitochondrial DNA B Resour.

[48] Zhao Q, Abuzeid AMI, He L, Zhuang T, Li X, Liu J (2021). The mitochondrial genome sequence analysis of *Ophidascaris baylisi* from the Burmese python (*Python molurus bivittatus*). Parasitol Int.

[49] Gao JF, Zhang XX, Wang XX, Li Q, Li Y, Xu WW (2019). According to mitochondrial DNA evidence, *Parascaris equorum* and *Parascaris univalens* may represent the same species. J Helminthol.

[50] Jabbar A, Littlewood DT, Mohandas N, Briscoe AG, Foster PG, Müller F (2014). The mitochondrial genome of *Parascaris*
*univalens*—implications for a "forgotten" parasite. Parasit Vectors.

[51] Liu GH, Nadler SA, Liu SS, Podolska M, D'Amelio S, Shao RF (2016). Mitochondrial phylogenomics yields strongly supported hypotheses for Ascaridomorph nematodes. Sci Rep.

[52] Hartwich G. Die Vorderdarmstrukturen, das Exkretionsystem sowie der Kopfbau der Ascariden und ihre taxonomische Bedeutung. Wiss Z-Martin-Luther-Univ. 1954;3:1171–212.

[53] Sprent JFA, Stone AR, Platt HM, Khalil LF (1983). Observations on the systematics of ascaridoid nematodes. Concepts in nematode systematics.

[54] Osche G (1958). Beiträge zur morphologie, okologie und phylogenie der Ascaridoidea (Nematoda); parallelen in der evolution von parasit und wirt [morphology, ecology and phylogeny of Ascaridoidea (Nematoda); parallels in the evolution of parasite and host]. Z Parasitenkd.

[55] Chabaud AG. Ordre des Ascaridida. In: Grassé PP, editor. Traité de Zoologie. Tome IV, fascicule 3. Paris: Masson et Cie; 1965. p. 932–1025.

[56] Nadler SA, D'Amelio S, Fagerholm HP, Berland B, Paggi L (2000). Phylogenetic relationships among species of *Contracaecum* Railliet & Henry, 1912 and *Phocascaris* Høst, 1932 (Nematoda: Ascaridoidea) based on nuclear rDNA sequence data. Parasitology.

[57] Sprent JFA (1990). Some ascaridoid nematodes of fishes: *Paranisakis* and *Mawsonascaris* ng. Syst Parasitol.

[58] Wolstenholme DR, Okimoto R, Macfarlane JL (1994). Nucleotide correlations that suggest tertiary interactions in the TV-replacement loop-containing mitochondrial tRNAs of the nematodes, *Caenorhabditis elegans* and *Ascaris suum*. Nucleic Acids Res.

[59] Wang BJ, Gu XB, Yang GY, Wang T, Lai WM, Zhong ZJ (2016). Mitochondrial genomes of *Heterakis gallinae* and *Heterakis beramporia* support that they belong to the infraorder Ascaridomorpha. Infect Genet Evol.

[60] Liu SS, Liu GH, Zhu XQ, Weng YB (2015). The complete mitochondrial genome of *Pseudoterranova azarasi* and comparative analysis with other anisakid nematodes. Infect Genet Evol.

[61] Park YC, Kim W, Park JK (2011). The complete mitochondrial genome of human parasitic roundworm, *Ascaris*
*lumbricoides*. Mitochondrial DNA.

[62] Chen Y, Wang L, Zhou X, Tang R, Li Y, Liu Y (2021). The mitochondrial genome of the sheep roundworm *Ascaris ovis* (Ascaridida: Nematoda) from southwest China. Mitochondrial DNA B Resour.

[63] Xie Y, Zhang Z, Wang C, Lan J, Li Y, Chen Z (2011). Complete mitochondrial genomes of *Baylisascaris schroederi*, *Baylisascaris ailuri* and *Baylisascaris transfuga* from giant panda, red panda and polar bear. Gene.

[64] Xie Y, Zhang Z, Niu L, Wang Q, Wang C, Lan J (2011). The mitochondrial genome of *Baylisascaris procyonis*. PLoS ONE.

[65] Jin YC, Li XY, Liu JH, Zhu XQ, Liu GH (2019). Comparative analysis of mitochondrial DNA datasets indicates that *Toxascaris leonin*a represents a species complex. Parasit Vectors.

[66] Zhou CY, Ma J, Tang QW, Zhu XQ, Xu QM (2021). The mitogenome of *Ophidascaris wangi* isolated from snakes in China. Parasitol Res.

[67] Zhao JH, Tu GJ, Wu XB, Li CP (2018). Characterization of the complete mitochondrial genome of *Ortleppascaris sinensis* (Nematoda: Heterocheilidae) and comparative mitogenomic analysis of eighteen Ascaridida nematodes. J Helminthol.

[68] Kijewska A, Rokicki J, Sitko J, Wegrzyn G (2002). Ascaridoidea: a simple DNA assay for identification of 11 species infecting marine and freshwater fish, mammals, and fish-eating birds. Exp Parasitol.

[69] Nadler SA, Carreno RA, Mejía-Madrid H, Ullberg J, Pagan C, Houston R, Hugot JP (2007). Molecular phylogeny of clade III nematodes reveals multiple origins of tissue parasitism. Parasitology.

[70] Zhu X, Gasser RB, Jacobs DE, Hung GC, Chilton NB (2000). Relationships among some ascaridoid nematodes based on ribosomal DNA sequence data. Parasitol Res.

[71] Honisch M, Krone O (2008). Phylogenetic relationships of Spiruromorpha from birds of prey based on 18S rDNA. J Helminthol.

[72] He X, Lü MN, Liu GH, Lin RQ (2018). Genetic analysis of *Toxocara cati* (Nematoda: Ascarididae) from Guangdong province, subtropical China. Mitochondrial DNA A DNA Mapp Seq Anal.

[73] Li Y, Niu L, Wang Q, Zhang Z, Chen Z, Gu X (2012). Molecular characterization and phylogenetic analysis of ascarid nematodes from twenty-one species of captive wild mammals based on mitochondrial and nuclear sequences. Parasitology.

