# Supplementary material for: Mitogenomic phylogenies suggest the resurrection of the subfamily Porrocaecinae and provide insights into the systematics of the superfamily Ascaridoidea (Nematoda: Ascaridomorpha), with the description of a new species of Porrocaecum
Source: Parasit Vectors. 2023 Aug 10;16:275. doi: 10.1186/s13071-023-05889-9 (PMC10416420; doi:10.1186/s13071-023-05889-9)
Supplement: Supplementary file 1 — Additional file 1: Table S1. The partitioning schemes and the optimal model selected for each combination of partition for the BI inference. [file 13071_2023_5889_MOESM1_ESM.docx]

| Subset | Best Model | Number of sites | Partitioning schemes |
| --- | --- | --- | --- |
| 1 | WAG+G | 198 | *atp*6 |
| 2 | CPREV+I+G | 760 | *cox*2; *cox*1 |
| 3 | CPREV+I+G | 1181 | *cox*3; *nad*1; *nad*3; *nad*5 |
| 4 | CPREV+G | 788 | *nad*2; *nad*6; *cyt*b |
| 5 | MTMAM+I+G | 452 | *nad*4L; *nad*4 |

**Table S1.** The partitioning schemes and the optimal model selected for each combination of partition for the BI inference.
